# Secondary Structure, a Missing Component of Sequence-Based Minimotif Definitions

**DOI:** 10.1371/journal.pone.0049957

**Published:** 2012-12-07

**Authors:** David P. Sargeant, Michael R. Gryk, Mark W. Maciejewski, Vishal Thapar, Vamsi Kundeti, Sanguthevar Rajasekaran, Pedro Romero, Keith Dunker, Shun-Cheng Li, Tomonori Kaneko, Martin R. Schiller

**Affiliations:** 1 School of Life Sciences, University of Nevada Las Vegas, Las Vegas, Nevada, United States of America; 2 Department of Molecular, Microbial, and Structural Biology, University of Connecticut Health Center, Farmington, Connecticut, United States of America; 3 Department of Computer Science and Engineering, University of Connecticut, Storrs, Connecticut, United States of America; 4 Center for Computational Biology and Bioinformatics, Indiana University School of Medicine, Indianapolis, Indiana, United States of America; 5 Department of Biochemistry, University of Western Ontario, London, Ontario, Canada; University of Alberta, Canada

## Abstract

Minimotifs are short contiguous segments of proteins that have a known biological function. The hundreds of thousands of minimotifs discovered thus far are an important part of the theoretical understanding of the specificity of protein-protein interactions, posttranslational modifications, and signal transduction that occur in cells. However, a longstanding problem is that the different abstractions of the sequence definitions do not accurately capture the specificity, despite decades of effort by many labs. We present evidence that structure is an essential component of minimotif specificity, yet is not used in minimotif definitions. Our analysis of several known minimotifs as case studies, analysis of occurrences of minimotifs in structured and disordered regions of proteins, and review of the literature support a new model for minimotif definitions that includes sequence, structure, and function.

## Introduction

Minimotifs provide an important piece of the mechanistic and theoretical basis for understanding protein-protein interactions and post-translational modifications, and thus the regulation of many cellular processes. Minimotifs are short contiguous peptide elements in proteins that mediate some biological function and are also called short linear motifs (SLiMs). In order to help standardize minimotifs, the Seefeld Convention developed a syntax to describe the amino acid sequence of a minimotif, which also encodes some specific types of chemical modifications [1]. This syntax had some limitations including a lack of standardized functional definition. Therefore, a new minimotif model was recently introduced which included a rich semantic syntax containing 22 attributes (later refined to have 29 attributes) [2], [3]. The basis of this model is a syntactical triplet comprised of the protein that contains the minimotif (*Source*), a description of the minimotif functionality (*Activity*), and the protein or molecule needed for the minimotif activity (*Target*). This triplet has properties unique to the triplet unit, as do each of the triplet elements.

The most pressing problem in understanding and identifying new minimotifs is the prediction of high numbers of false positives based on sequence analysis. This is thought to be primarily due to the low complexity of the protein sequence-based definitions, where such sequences can occur frequently in proteomes by random chance. For example, there are more than 18,000 YxN sequences in the human, rat and mouse proteomes that are predicted to bind to the Grb2 SH2 domain. There have been a number of efforts to reduce these false positive predictions. The most successful thus far is a data-driven approach that uses other relationships such as protein surface location, protein-protein interaction, and cellular function to reduce false positives in a trained linear regression or neural network algorithm [4]–[7].

Although these data-driven approaches for reducing false positives continue to improve, they do not address the fundamental problem of the minimotif definition: some sequences that match a minimotif consensus sequence are functional, while others are not. This general observation indicates that there are shortcomings to the sequence definition itself that do not capture the true specificity of interactions that are observed in cells.

A clue to a potential deficiency in minimotif definitions comes from a number of observations concerning secondary structures of minimotifs bound to their targets. Protein secondary structures can generally be classified as follows, with single letter codes for individual elements taken from the Dictionary of Protein Secondary Structure (DSSP): helices [α–helix (H), π-helix (I), 3–10 helix, 2–7 helix, polyproline helix, and collagen helix], β-strands that hydrogen bond to form β-sheets [β-strand or β-bulge (E)], and a series of turns [α-turn, β-turn, γ-turn, δ-turn, and π-turn (T), with random coil (C) as an additional category] [8], [9]. Each category may have many subtypes as exemplified by the β-turn, which has nine different subtypes with differing φ and ψ angles for each amino acid [9]. Minimotif sequences are found in many of the known secondary structures including α-helices, β-strands, and turns, with a set of examples shown in **Table 1** and **Fig. S1** [10]. Examples of helix minimotifs include an α-helix motif that binds calmodulin and a polyproline helix minimotif that binds SH3 domains [11]–[14]. Examples of β-strand minimotifs include the common theme of β-addition, such as that observed in PDZ and PTB domains where a protein's existing β-sheet is paired with a minimotif in a β-strand, thus extending the β-sheet [15]. There are also a number of different types of turn motifs that are involved in minimotif recognition. One example is a β-turn in elastin that binds laminin [16]. Since proline residues are enriched in turns, proline seems to be exploited as a determinant in minimotifs [17]–[19].

**Table 1:** Examples of Minimotifs with known secondary structure.

Secondary structure	Minimotifs (function)	References*
**Helices**		
α–helix (H)	Mettelin binds Calmodulin	[107]
π-helix (I)	unknown	
2–7 helix (coiled coil)	Leu zipper dimerization in C/EBP4	[108]
3–10 helix (G)	Gab2 binds Grb2 SH3 domain; SLP-76 binds SH3 domain of Gads	[51], [109]
polyproline helix	SH3, WW, PX, EVH1 domains	[11], [12], [14], [19], [52], [56], [89], [95], [110]–[116]
collagen helix	collagen binds integrins	[28]
**β-strands**		
β-strand (E)	APP binds Dab 1	[117]
**Turns**		
α-turn (T)	IGFBP1 binding peptide	[118]
β-turn (T)	Elastin binds lamininFe65L1 binds APPYxN (Grb2 SH2)	[16], [43], [44], [119]–[122]
γ-turn (T)	HIV Protease	[123], [124]
δ-turn (T)	unknown	
π-turn (T)	unknown	
bend (S)	unknown	
Random coil (C)	unknown	

* references include other minimotifs that are known to be in this secondary structure.

The current minimotif definitions include sequence and function [2]. Here, we consider whether or not the minimotif definition needs to also include structure. In addition to the analyses presented herein, a case study for various minimotifs with the sequence RGD that bind to different Integrin heterodimers shows why structure must be considered for inclusion in minimotif definitions. Eight vertebrate Integrin subunits form α/β heterodimers that bind extracellular matrix protein ligands containing the RGD sequence [20]. Several RGD conformations are important for Integrin binding specificity [21], [22]. Integrin α_2b_/β_3_ binds to RGD sequence in a type II β-turn, but not to peptides that have a type I or III β-turn [23]. The α_v_/β_3_ and α_v_/β_5_ Integrins bind to RGD ligands in a type IV β-turn, whereas α_2b_/β_3_ RGD ligands are thought to bind in a type II′ β-turn [24]–[26]. Synthetic mimetics of α_2b_/β_3_ integrin have RGD ligands in a γ-turn [27]. α_2_/β_1_ and likely α_1_/β_1_, α_10_/β_1_, and α_11_/β_1_ Integrins binds to the RGD motif in a collagen triple helix [28]. Understanding the structure of RGD ligands is important as RGD mimetics such as Eptifibatide are therapeutically used as platelet aggregation inhibitors [29]. Eptifibatide is a cyclic RGD-containing heptapeptide that has a distinct ligand binding conformation (2VDN) [20]. The RGD minimotif sequence definition is thus ambiguous, unless it is deconvolved using a revised definition that also includes structure.

Further support for including structure comes from our analysis of the binding of the YxN sequence to the Grb2 SH2 domain presented herein. This is one of the best-studied minimotif sequences, with multiple randomized library screens and multiple solved structures. Grb2 is an adaptor protein involved in growth factor signaling and also has several other functions [30]. The SH2 domain of Grb2 binds to the consensus sequence YxN (single letter amino acid code), where Y represents a tyrosine that must be phosphorylated, x is any of the 20 amino acids, and N represents asparagine.

Our analysis revealed that the structures of less than 1% of YxN sequences in the PDB are in the β-turn configuration that is recognized by the Grb2 SH2 domain. Unless the other ∼99% of YxN instances in the PDB having other structures can morph into a β-turn, these ligands are not physically capable of binding the YxN ligand binding site in Grb2 with a reasonable affinity. In this paper we provide evidence to support a new minimotif model that includes structure, which will undoubtedly help to resolve the long-standing problem of minimotif specificity.

## Results

### Problems with minimotif sequence definitions

We wanted to study if structures should be used in minimotif definitions, but first needed to address a problem with the minimotif sequence definitions. Currently, sets of minimotif instances are interpreted by producing consensus sequences that reflect identifies and similarities at each position in the minimotif. For instance, [ST]xx[DE] is a typical consensus sequence expression found in substrates phosphorylated by Casein Kinase II [31]. This expression is ambiguous, an overinterpretation of the experimental data, and represents a significant loss of information compared to the known instances of proteins that are phosphorylated by Casein Kinase II. One source of minimotif definition ambiguity is that consensus definitions do not capture the probability of each amino acid at each position—an amino acid occurring only one time in 20 instances could be included in a sequence definition or left out, depending on the discoverer's preference. This problem is solved by the use of position specific-scoring matrices (PSSMs) that define the probability of each amino acid at each position.

Despite their advantage over simple consensus sequences, PSSMs also still suffer from ambiguity, overinterpretation, and loss of information. Consider the [ST]xx[DE] minimotif as an illustrative example. What does the expression [ST]xx[DE] mean? The bracketed portions imply that this minimotif could encode SxxD, TxxD, SxxE, and TxxE. There is no way to use this regular expression to determine which of the four expressions are valid. If SxxD and TxxE are the only valid consensi, the [ST]xx[DE] regular expression is an overinterpretation. Similarly, the “xx” in the middle of the regular expression implies that all 400 permutation of this pair of residues have been tested and verified, which is most often not the case, and is thus another source of overinterpretation.

The other major problem with these types of definitions is the loss of string information. While scientists routinely present minimotifs as 1-dimensional sequence strings, these are chemical peptides with well-defined 3-dimensional structures when bound to a target (**Fig. S1**). There are clear interdependencies of positions in short minimotif structures. The existing minimotif syntax implicitly assumes all positions are independent of each other. For instance, in the “xx” part of the aforementioned consensus sequence, it is not just important to know that there are two amino acids, but which of the 400 possible combinations of amino acids are valid.

Despite these problems with the consensus sequence approach to minimotif definition, nearly all reports of minimotifs currently use this methodology in practice.

### Grb2 SH2 binding minimotif as a model for investigating minimotif structure

In order to accurately present minimotifs in this study we have explored a lexical set of all possible permutations of a minimotif. The lexical set definition overcomes the problems of ambiguity, loss of string information, and overinterpretation present in consensus sequences and PSSMs. Furthermore, we have assessed whether structure should be included with the lexical set as part of the minimotif definition. To this end, we have first investigated the YxN minimotif that binds to the Grb2 SH2 domain. This minimotif was chosen as a model because it relatively simple, has had multiple studies that have investigated its specificity, and has a number of structures of the target domain bound to the minimotif source. In its simplest form, all studies have identified the consensus minimotif as YxN, where the tyrosine residue is phosphorylated.

Grb2 is known to interact with ∼29 proteins through this minimotif (**Table S1**). There are ∼18,000 YxN instances in human proteins, indicating an over prediction of valid occurrences by several orders of magnitude. In fact, most minimotifs exhibit similar levels of overprediction; YxN is thus a representative example. The vast amount of data for this minimotif afforded us the opportunity to study why there is such poor predictive capability for minimotif consensus sequences.

**Figure 1:**
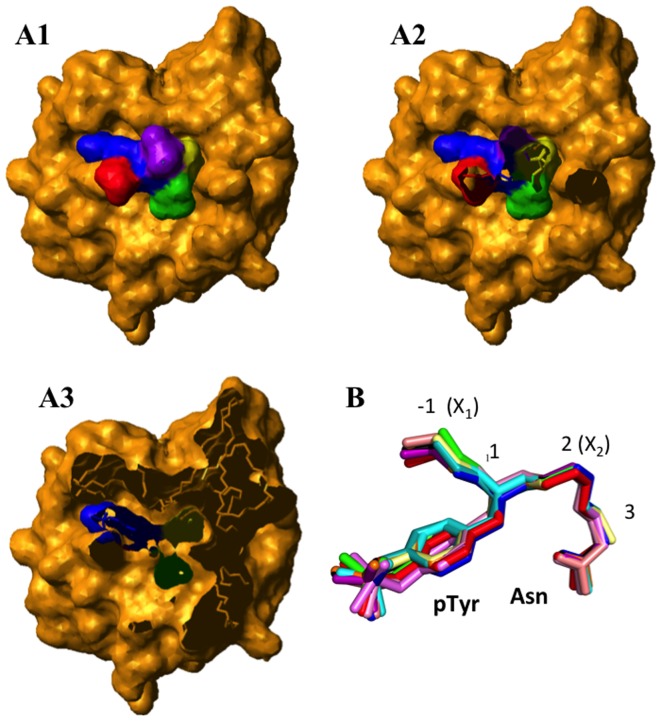
Structurally conserved xYxN minimotif bound to the Grb2-SH2 domain. A. Surface plot of the Grb2-SH2 domain bound to a tyrosine-phosphorylated Shc1 peptide (1JYR). The SYVN Shc1 peptide is colored: S (red), Y (blue), V (green), N (yellow); these are the only four residues that make contact with the SH2 domain (gold). The C-terminal V residue in the SYVNV peptide is colored purple and does not contact the SH2 domain. Three different cut-away planes are shown in A1–A3. B. Alignment of structures of peptides when bound to the Grb2 SH2 domain—Grb2-SH2 domain binds to a conserved 3D structural β-turn motif. Peptides are 1BMB (black), 1FYR (blue), 1JYR (orange), 1TZE (purple), 1BM2 (red), 2H5K (cyan), 3N7Y (violet), 3N8M (green), and 3N84 (pink), 1ZFP (salmon), 2B3O (pale yellow), 2SHP (teal), 1QG1 (olive), and 3KFJ (brown). Backbone RMSD for 14 peptides = 0.4 Å average, with a maximum of 1.1 Å. The conserved Asn and pTyr side chains are shown. Numbering of residues is relative to P-Tyr in the +1 position.

We first examined if any other residues besides the YxN make contact in structures of Grb2 complexes with YxN minimotifs. The −1 residue (relative to the phosphotyrosine) also made contact with Grb2, so the sequence definition was expanded to xYxN for further evaluation (**Fig. 1A**). The xYxN peptide ligands in 14 separate structures of this minimotif bound to the SH2 domain of Grb2 were structurally aligned. All structures of xYxN when bound to Grb2 were well conserved with an average RMSD of 0.4 Å for backbone and C_β_ atoms; C_β_ atoms were included to better define the overall orientation of side chains. An alignment of these minimotifs is shown in **Fig. 1B**. This result indicates that the minimotif ligand in the Grb2-SH2 complex has a conserved structure.

### Which xYxN sequences naturally occur in the correct Grb2 SH2 β-turn ligand structure?

We wanted to use experimental data to determine which xYxN sequences can form the Grb2 SH2 β-turn ligand structure. The PDB contains ∼81,000 structures, providing a rich source of structural information for xYxN sequences. A sequence search of the PDB reveals ∼57,400 structures with the xYxN sequence; if the 400 xYxN lexica were randomly distributed, we can assume an average sampling of ∼140 instances of each lexicon. Although the PDB is not a random sample, it can be used to determine which xYxN lexica form the β-turn ligand.

**Figure 2:**
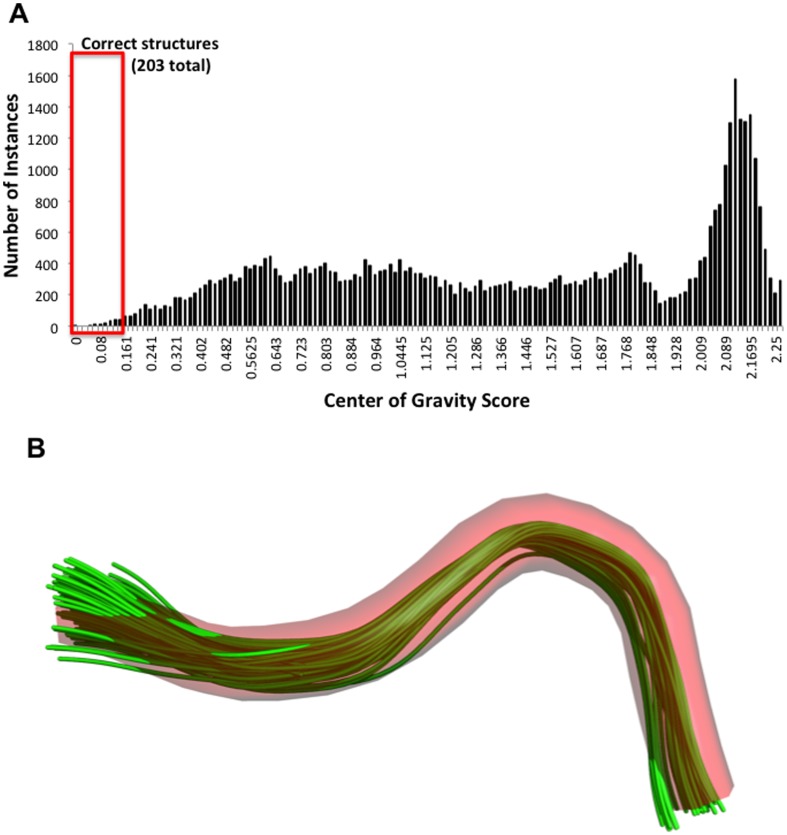
Identification of xYxN sequences in the PDB with the same structure as Grb2 SH2 ligands. A. A bar plot showing the distribution of centroid scores for the 46,593 xYxN structures in the PDB fit to the structure of the Grb2 SH2 peptide ligand (1JYR). The red box shows the 203 structures that match the known positive minimotifs. B. A fit of the C_α_ backbone traces of the 203 matched structures (green lines) to the backbone trace of the xYxN structure in 1JYR (red pipe).

A Centroid Algorithm was used to fit, score, and rank the similarity of the backbone and C_β_ atoms of the xYxN ligand in the 1JYR structure with 46,593 of 57,400 xYxN structures in the PDB for which complete structural data exists [32]. C_β_ atoms are included to help define the orientations of the side chains. The distribution of centroid scores for the 46,593 structures ranged between 0.002 and 13.7 (**Fig. 2A**). For the 14 known structures of the xYxN minimotif bound to Grb2, the centroid scores ranged between 0.002 and 0.142, providing a measure of the variability in the minimotif structure. We used a threshold that was 10% higher than the maximal value to ensure that our search did not miss potential positives. Applying this threshold score of 0.16 produced 203 structures that had xYxN sequences in the correct β-turn and with correct orientation of the C_β_ atom in the side chain (**Fig. 2B**).

**Figure 3:**
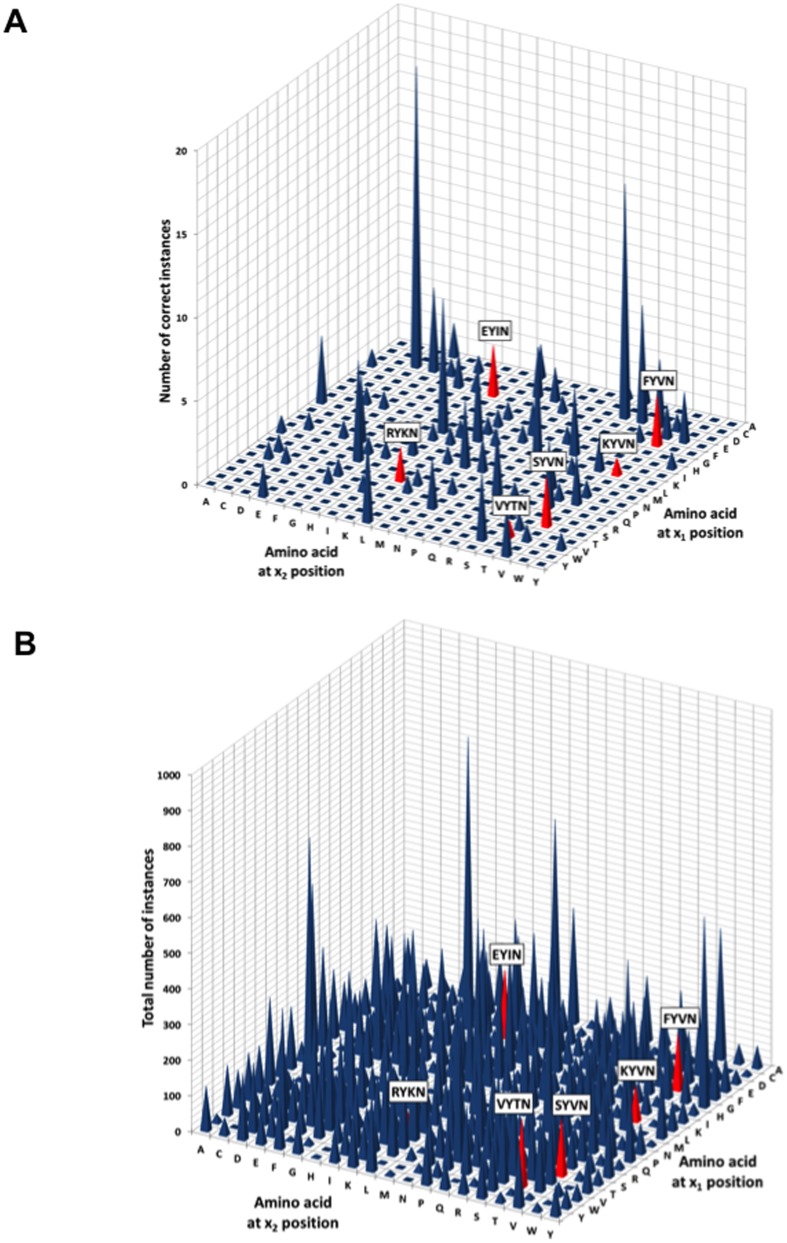
Frequencies of xYxN lexica and those lexica in a β-turn structure in the PDB. A, B. Plot of the number of occurrences of each xYxN lexicon for a β-turn (A), or total number in the PDB (B). The depth and horizontal axes shows the single letter IUPAC code for amino acids in the X_1_ and X_2_ positions of the xYxN consensus minimotif, respectively. Colored labeled bars indicate lexica where a known structure of a complex of the Grb2 SH2 domain with this peptide sequence exists in the PDB.

These structures were encoded by 91 of the 400 possible xYxN lexical sequences (**Figs. 3** **and S2**). While on average each sequence was sampled in the PDB ∼140 times, 396 of the 400 permutations were observed at least one time in the PDB (**Fig. 3B**). The normalized frequency of occurrence of each xYxN lexical sequence in the PDB with respect to the correct β-turn structure is shown in **Fig. S3**. We conclude that only ∼23% of the 400 xYxN sequence permutations are observed in the correct β-turn ligand conformation in the structures from the PDB.

We reviewed the literature and identified 29 known positive xYxN sequences that bind to the Grb2 SH2 domain (**Table S1**). Of these, 90% were identified as a sequence known to form a β-turn from our analysis of the xYxN structures in the PDB. This is a vast improvement over the xYxN consensus sequence definition used without considering known structures. This new approach to minimotif definitions resolves the ambiguity and loss of string information present in consensus sequences and PSSMs. Since this result reduces the number of lexica in the xYxN minimotif definition ∼4-fold (23% of lexica were in the correct structure) and since the identified xYxN lexica were consistent with known positives, the data suggest that structure should be included in minimotif definitions.

### Analysis of the secondary structures of xYxN minimotif sequences

**Figure 4:**
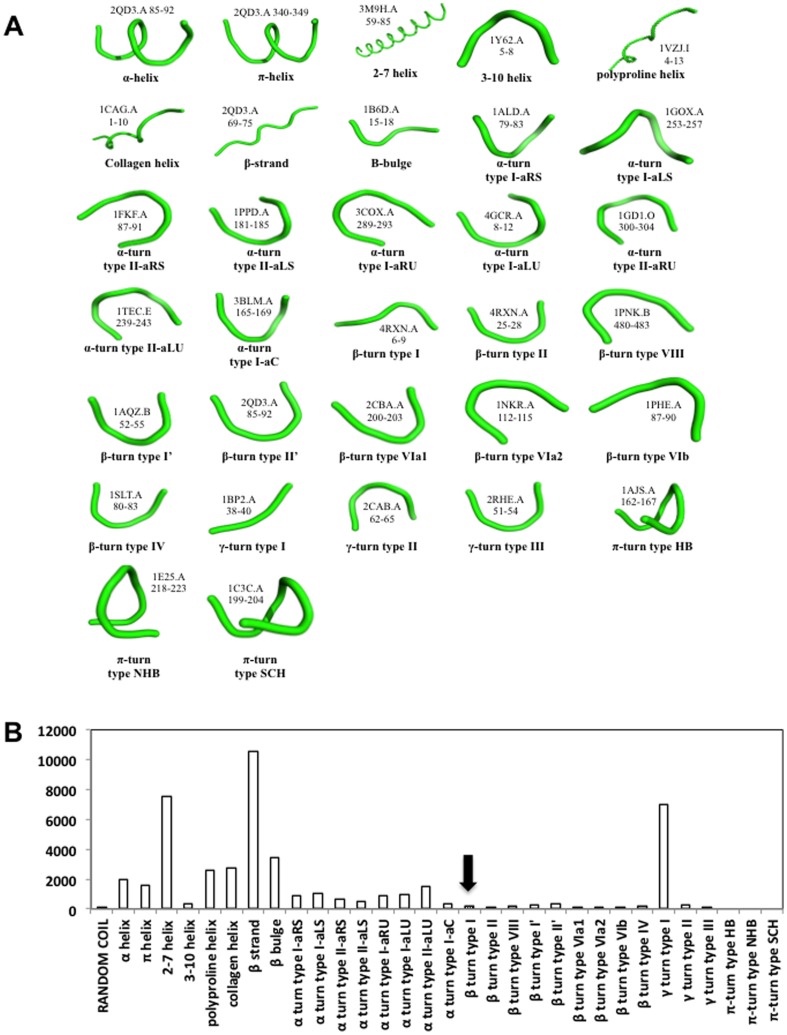
Minimotif secondary structure folds and distribution of xYxN folds. A. Images of 32 secondary structures. The structure, chain, and residue range for each type of secondary structure are shown. B. Bar graph showing the number of occurrences of xYxN sequence matches from the PDB in each type of secondary structure. Unfilled bar indicates the correct structure of known ligands. Arrow indicates the structure of the known Grb2 ligand.

Since such a small portion of the xYxN sequences were in a β-turn, we examined the prevalence of this minimotif in other types of secondary structures in the PDB. The β-turn is only one of many types of secondary structures, so we first determined the different types of secondary structures. The DSSP has several secondary structures, but does not have a complete list of current secondary structures. A review of the literature identified the 32 secondary structures, shown in **Fig. 4A** (there is an additional δ-turn secondary structure, but no examples were provided in the literature). We also include a category of random coil to collect structures that do not fit into these 32 categories.

A similar structure-search approach was used to examine the prevalence of each of the 32 secondary structures formed by the xYxN sequences in the PDB. Of the 32 secondary structures, xYxN was rarely observed in 13 structure types, moderately observed in 15 structure types, and frequently observed in 2–7 helices, β-strands, and type 1 γ-turns (**Fig. 4B**). Only 0.4% of the 46,593 occurrences of xYxN in the PDB were in the correct experimentally determined type I β-turn configuration of the Grb2 xYxN ligand (**Table 2**).

**Table 2:** Statistics for percentages of lexica with minimotif secondary structures.

Minimotif consensus	Binding domain	Secondary structure	PDB ids	# lexica	Instances in PDB	# lexica in PDB	# structured lexica in PDB	# structured instances in PDB	% structured lexica	% structured instances
xYxN	SH2	β-turn	1BM2, 1BMB, 1FYR, 1JYR, 1QG1, 1TZE, 2AOB, 2H5K, 2HUW, 3IMD, 3IMJ, 3IN7, 3IN8, 3KFJ, 3N7Y, 3N84, 3N8M	400	46593	396	91	203	23%	0.44%
xx[ST]x[IVL]>	PDZ	β-strand	2I04, 2KPL, 3NFK	48000	2926	690	257	257	37%	8.8%
[ILV]QxxxRGxxx[RK]	Calmodulin	α–helix	1IWQ, 1MXE, 1N2D, 2BCX, 2IX7, 2K0F, 2KXW, 2L53, 2L7L, 2UX0, 2VAS, 3GOF, 3HR4, 3OXQ	3.84×10^8^	73	24	5	10	21%	13.7%
[RK]xxK	SH3	3–10 helix	1H3H, 1OEB, 1UTI, 2DON	800	104100	794	248	797	31%	0.008%
PxxPxK	SH3	Polyproline helix	1CKA, 1CKB	8000	3923	549	17	39	3%	1.0%
IxxNT	Coiled coil	2–7 helix	2WQ0, 2WQ1, 2WQ2, 2WQ3	400	5578	276	66	378	24%	6.8%

>indicates that the motif must be on the C-terminus.

### Other minimotif definitions are more precise when structure is added

We questioned whether other minimotifs were like the xYxN minimotif by examining if inclusion of secondary structure helped to refine the minimotifs definitions. We selected a representative set of five additional minimotifs having differing types of ligand secondary structures (β-strand, α-helix, 3–10 helix, 2–7 helix, and polyproline helix). A summary of results for structural similarity of these minimotifs is shown in **Table 2**. As observed for the Grb2 minimotif, these minimotifs were more often observed in secondary structures that did not match the structure of the known positive minimotif ligands (**Table 3** **, Fig. S4**). These analyses show that for six different minimotifs, the percentage of instances in the PDB with the correct structure ranges from 0.008% to 13%, with an average of 5%. The percentage of lexical permutations with at least one structure in the correct minimotif structure ranges from 3%–37% with an average of 23%. In the most stringent case, only 3% of the 8000 PxxPxK minimotif lexica for binding the Crk SH3 domain were observed in the correct polyproline helix structure, suggesting that structure is likely a critical component of this minimotif definition.

**Table 3:** Statistics for structure of minimotifs in the PDB.

Secondary structure	xYxN^1^	IxxNT^1^	[RK]xxK^1^	PxxPxK^1^	xx[ST]x[IVL]>^1^	[ILV]Qxxx RGxxx[RK]^1^	Total^2^
**α helix**	1966	32	5257	2	51	**0**	**7308**
**π helix**	1549	19	2974	0	7	0	**4549**
**2–7 helix**	7544	**378**	21230	29	220	0	**29401**
**3–10 helix**	377	48	**797**	216	261	10	**1709**
**polyproline helix**	2622	1	3774	**39**	91	0	**6527**
**collagen helix**	2755	0	5366	31	40	0	**8192**
**β strand**	10557	1	15579	26	**257**	0	**26420**
**β bulge**	3429	9	4407	550	327	3	**8725**
**α turn type I-aRS**	868	14	1609	17	4	1	**2513**
**α turn type I-aLS**	1023	1	2605	0	1	0	**3630**
**α turn type II-aRS**	680	0	747	1	2	0	**1430**
**α turn type II-aLS**	488	2	928	1	6	0	**1425**
**α turn type I-aRU**	906	0	1488	1	1	2	**2398**
**α turn type I-aLU**	995	0	993	1	1	0	**1990**
**α turn type II-aLU**	1522	0	1690	0	0	0	**3212**
**α turn type I-aC**	378	0	439	4	3	0	**824**
**β turn type I**	**203**	33	236	173	178	5	**828**
**β turn type II**	117	6	165	37	58	1	**384**
**β turn type VIII**	214	0	176	82	16	0	**488**
**β turn type I′**	290	172	591	240	258	10	**1561**
**β turn type II′**	369	15	777	156	53	0	**1370**
**β turn type VIa1**	25	0	14	20	0	0	**59**
**β turn type VIa2**	14	0	41	12	0	0	**67**
**β turn type VIb**	105	0	71	33	14	0	**223**
**β turn type IV**	184	36	256	88	33	0	**597**
**γ turn type I**	7016	676	10431	804	770	22	**19719**
**γ turn type II**	269	182	639	216	185	16	**1507**
**γ turn type III**	101	49	90	118	46	3	**407**
**π turn type HB**	0	0	3296	1	9	0	**3306**
**π turn type NHB**	0	8	2377	1	9	0	**2395**
**π turn type SCH**	0	13	4621	0	25	0	**4659**
**Random coil**	26	3883	10436	1024	0	0	**15369**
**Total**	**46592**	**5578**	**104100**	**3923**	**2926**	**73**	**163192**

1 Values do not include known positives used for search.

2 Total column is the sum of the six minimotifs.

These results are, on average, similar to those observed for the Grb2 β-turn ligand. These results further support our contention that structure should be included in minimotif definitions. This analysis also demonstrates that if structure is included in a minimotif definition, a substantial portion of lexica (average = 77%) are never observed in the correct structure, thereby, their elimination results in a large increasing the specificity of the minimotif definition.

### Are all minimotifs structured or disordered?

There have been a number of reports that minimotifs are concentrated in disordered regions of proteins [33]–[39]. However, this conclusion is not based on analysis of a large number of diverse types of minimotifs. We therefore analyzed 245,000 minimotifs from the Minimotif Miner 3.0 database that matched protein sequences in known proteins using the PONDR VLXT neural network algorithm for disorder prediction [40]. Minimotifs were categorized as being completely in folded regions (structured), completely in disordered regions (unstructured) or with sequences having some segments ordered and some disordered (hybrid). Analysis of ∼245,000 minimotifs produced scores for ∼242,000 motifs; the remaining 3,000 motifs were incapable of being analyzed by the PONDR VLXT algorithm, primarily because the algorithm requires that protein segments be at least 30 amino acids long.

**Figure 5:**
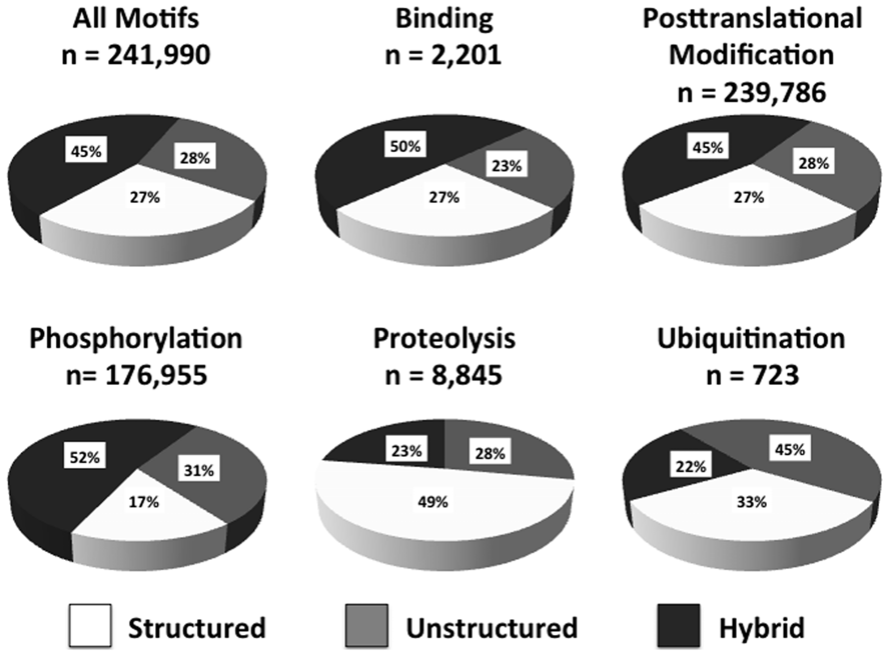
Pie graphs showing percentage of predicted order/disorder statistics for minimotifs. Results from analysis of ∼242,000 known minimotifs for all minimotifs tested, the subset of minimotifs with binding activities, and the subset of minimotifs with posttranslational modification activities, regarding their location in structured, unstructured, or both (hybrid) regions of proteins, as predicted by the PONDR VLXT algorithm. Also shown are similar results for phosphorylation, proteolysis and ubiquitination minimotifs. Percentages and total number of minimotifs in each category are shown.

From the analysis of the 242,000 minimotifs, 28% were unstructured, 27% were structured, and 45% were hybrid (**Fig. 5**). When segregated into minimotif types, there were 2,201 binding motifs, of which 23% were unstructured, 27% were structured, and 50% were hybrid. Modification minimotifs, with 239,786 motifs total, were 28% unstructured, 27% structured, and 45% hybrid. Similar results were obtained using the VSL2b algorithm [41]. Considering that the accuracy of these algorithms is estimated to be ∼85% [41], these results indicate that, even though minimotifs are structured when engaging their targets, some can exist in both disordered or ordered forms prior to engaging their targets.

We next sought to determine if specific minimotif activities had preferences for structured or unstructured regions of proteins. We focused our attention on those categories that had at least 75 known instances. Different minimotifs had vastly different preferences with regard to their location in structured or unstructured regions (**Fig. 5** and **Fig. S5**). N-glycosylation, lipidation, sulfonation, oxidation, and trafficking minimotifs, as well as proteolytic sites all had a much higher tendency to be in structured regions of protein (49–80%). Other types of minimotifs, including those for phosphorylation, hydroxylation, methylation, trimethylation, and O-glycosylation, all had a stronger preference for unstructured regions (46–80%). We also observed that most subcategories had a significant percentage of hybrid minimotifs (8–43%). These hybrid motifs, which have some amino acids that are structured and some that are unstructured, may be prone to an induced-fit type of interaction and presents an interesting topic for future investigation. In conclusion, different types of minimotifs are more so associated with structured or unstructured regions of proteins and support our contention that at least a significant portion of minimotifs are in both structured and unstructured regions of proteins prior to engaging the target; they are both structured once the target is engaged.

### Structure-based minimotif definitions and model

**Figure 6:**
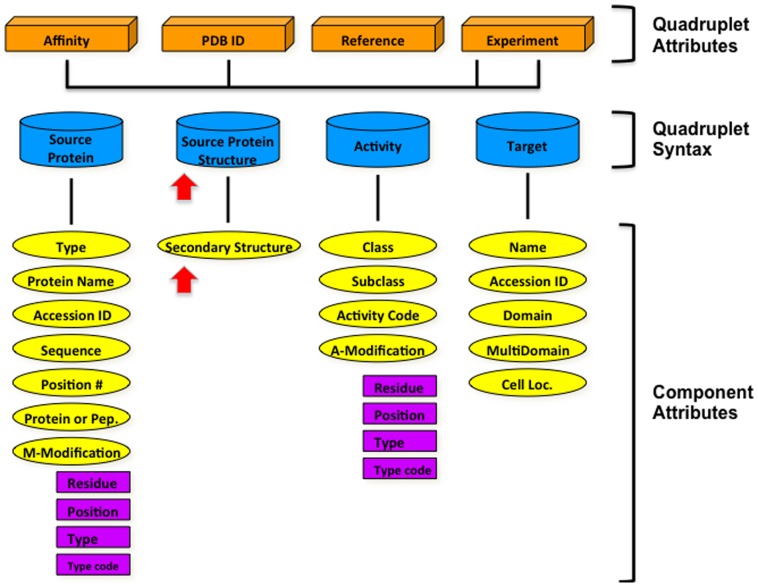
A generalized model of minimotifs that include structure in the minimotif definitions. Structure was added to the previously described minimotif model [2]. The structured syntax has a sequence and structure of the minimotif in the source protein, an activity, and a target that is associated with the minimotif activity (blue). This syntax quadruplet has properties such as an affinity, structure id, reference to the paper of discovery, and experimental support (orange). Each component of the syntax quadruplet has its own attributes (yellow). Two attributes have additional metadata. Red arrows indicate new features added to the model.

We had previously modeled the syntactical triplet of the minimotif source, activity, and target [2]. The minimotif analyses herein demonstrate that structure is a critical component of the definition of minimotifs and indicate that sequence alone is not sufficient to define a minimotif. This is likely to be a major contributor to the majority of false positive predictions. Based on these observations and supporting literature addressed in the discussion, we now propose a new model for minimotif definitions that includes sequence, structure, and function (**Fig. 6**). The new model is centered on a quadruplet that includes a chemistry definition (protein sequence and its chemical modifications) for a source protein, a structure of the minimotif in the source protein, an activity, and a target molecule.

We propose that the minimotif sequence definitions be revised to lexical sets with structure definitions to overcome the three aforementioned shortcomings of consensus sequences and PSSMs. Modifying the sequences to contain lexical sets is now trivial given current computational power.

The challenge is how to include structure in the model. When we initially defined a minimotif, we selected a length of 15 residues or shorter based on the idea that a minimotif is restricted to one secondary structure element. We first considered using the DSSP library of secondary structures or the BRiX database of structural units of short peptide segments observed in the PDB [42]. Ultimately, we decided to assign each motif to one of the 32 secondary structures given in **Fig. 4A** using the Centroid Algorithm because the vast majority of instances in the PDB fit one of these categories. Although the random coil category is heterogeneous, it represents a small percentage of the structures in the PDB (**Table 3**) and we have yet to identify a minimotif with a structure in the random coil category. We suggest this approach because the DSSP library is too general and could result in ambiguity. While the BRiX database would be comprehensive, it is likely not necessary, less intuitive, and would have a higher overhead limiting its use. Therefore, we propose that all minimotifs now contain a secondary structure identifier that defines a backbone structure for the minimotif.

The new minimotif definition should be of the form: The {sequences} set of sequences in the {secondary structure} secondary structure {activity} {target domain} of {target}.

## Discussion

Minimotifs are important functional elements in proteins that are often predicted based on sequence matches to consensus sequences or ranking using PSSMs. These approaches produce significant false-positives, limiting the usefulness of minimotif research. Although many types of data-driven approaches have been used to reduce false-positive predictions, the high number of false positives indicates that there is a missing theoretical element necessary to explain the specificity of minimotif activities observed in cells.

The original attempt at standardization of minimotifs helped to standardize protein sequence representation and include some of the known posttranslational modifications to minimotifs [1]. Our group has extended this syntax to include both standardized sequence, with its modification(s), and functions in the definitions [2]. In this paper we identify minimotif structure as a critical missing component in minimotif definitions and propose that minimotif definitions now include sequence, structure, and function. Our analysis of several known minimotifs as case studies, analysis of occurrences of minimotifs in structured and disordered regions, and review of the literature support this new definition model.

### What evidence justifies the inclusion of structure in minimotif definitions?

In the early 1990's it was recognized that structure is important in recognition of protease cleavage minimotifs, where those minimotifs present in a β-turn were processed, while similar minimotif sequences present in β-sheets and α-helices were not [43], [44]. Despite this observation, consensus sequences and PSSMs became the standard used to describe and study minimotifs. Several pieces of evidence justify our proposed addition of structure to minimotif definitions:

1. Current theory for minimotifs does not explain the specificity observed in cells. The example addressed in our analysis of the Grb2 SH2 domain-binding minimotif is typical of many consensus and PSSM motif definitions, where many false positives are predicted. After more than two decades of research by numerous labs it is clear that some fundamental determinant that drives the specificity is missing. Here, we propose this is structure.
2. Minimotif definitions are heterogeneous and ambiguous in the absence of structure. The example of interaction of RGD ligands with different integrin complexes presented in the Introduction shows how structures of minimotifs can encode additional specificity that cannot be captured with the minimotif sequence alone. Our analysis of the six example binding minimotifs shows that only ∼5% of the occurrences in the PDB and ∼23% of the lexica can be observed in the correct structure of the ligand. The addition of structure helps to refine those lexical sequences that bind the target.
3. There are many examples of minimotifs with structure. Hundreds of minimotifs in PEPX, 3DID, SLiMDiet, PeptiDB, and MnM databases have specific known structures [45]–[50]. There are many reports where minimotifs assume one of several common secondary structures [51]. For example, turns and polyproline helices are almost exclusively located on the protein surface where minimotifs interact with their targets [52]. Thus, it makes sense than a number of minimotifs are located in different types of turns. A structure filter has been implemented as an approach to reduce false positives in the Eukaryotic Linear Resource (ELM) server for predicting minimotifs [53]. Although ELM has implemented a minimotif disorder filter based on a disorder prediction algorithm, and has concluded that minimotifs are concentrated in disordered regions, their ROC curves and other validation analyses of their new structural filter shows that structures are indeed an important determinant for at least a significant fraction of minimotifs [37], [53], [54]. Further support for structured minimotifs comes from the study of synthetic secondary structure mimetics that can disrupt protein-protein interactions [55].
4. Many minimotifs are found in globular domains and have structure. The PDB web system has a query system that can be used to identify structures that contain a specific posttranslational modification or a site that is modified. These structures have hundreds of phosphorylation sites, ∼41 hydroxyprolines, ∼71 sulfotyrosines, and many other types of modifications. Furthermore, there are many proteins that have structured protease sites and N-glycosylation sites. All of these minimotifs have proteins or protein domains with structures. Further support for the presence of minimotifs in globular domains comes from an analysis of the MnM and ELM minimotif databases with SLiMDiet, which found many minimotifs located in globular domains, despite the poor coverage of proteins with known structure in the PDB [45]. One example of a binding minimotifs in a domain is the p47phox SH3 domain, which has an intramolecular interaction with a polyproline minimotif in its PX domain [56]. Analysis of these databases also shows minimotifs binding to the surface of the protein, a fact taken advantage of in the surface prediction filter of MnM [48]. Our global analysis of ∼242,000 minimotifs does suggest that many minimotifs are structured, disordered, or hybrid motifs with both structured and disordered regions. This global analysis indicates that minimotifs are not concentrated in disordered regions of proteins as previously thought.

### Disambiguated Disorder

Many scientists have concluded that minimotifs are unstructured and highly concentrated in disordered regions or regions of intrinsically unstructured proteins (IUPs); these are also called intrinsically disordered proteins (IDPs) [37], [53], [54], [57]–[61]. While this idea seems to be in conflict with structured minimotifs, this disparity may arise from the ambiguous definitions of IUP and related terms. IUP and IDP are misnomers because numerous studies show that IUPs have significant secondary structure, which is supported by some definitions, but not others [58], [62]–[66]. Secondary structures are ordered structures that can exist in the absence of any tertiary structure. Thus, IUPs likely have an intermediate level of order when compared to globular domains and completely unstructured random coiled proteins. We think that “a two-state models where each residue is either ordered or disordered” is not sufficient to explain the different degrees of order and disorder that are observed in peptides and IUPs [58].

Clearly, terms are needed to distinguish these different levels of disorder/order. Here we consider that IUPs lack a distinct, stable tertiary structure, but are constructed of a set of secondary structures that may be either stable, or sample various secondary structures on different temporal timescales. These timescales likely range from milliseconds to picoseconds [67]–[70].

With regard to this disambiguated definition of IUPs, the lack of structure of minimotifs in IUPs reported by others needs to be reevaluated. There are three possibilities. One possibility is that a minimotif may be in a region of an IUP that contains no secondary structure and nucleates into a defined structure when it interacts with its target as has been previously proposed [58], [71]–[73]. Our analysis of ∼242,000 minimotifs suggests that the majority of minimotifs are in regions that have some order and some disorder, which would be consistent with this induced fit model. However, the disorder prediction algorithms do not accurately predict the exact residues where disorder begins or ends. There are more than a dozen algorithms used to predict IUPs in the proteome, but these do not rigorously test if these regions contain any secondary structure. Dunker et al. note that IUPs can contain secondary structures [74]. We must consider that there are some isolated cases where a lack of secondary structure in a minimotif has been validated, e.g. [75]; however, the evidence supporting this hypothesis on a global scale is by no means conclusive. Such conclusions are based largely on computer-based predictions that have relatively high intrinsic error rates and do not rigorously assess the presence of ordered secondary structures.

Since disordered segments are dynamic, it is possible that even if a protein were completely disordered, a transient structure could be recognized by a binding partner or modification enzyme. If ∼1% of an IUP has a secondary structure at any given time, this would not be detectable by current techniques that assess the average structure of a population of molecules. If 1% of a protein had secondary structures, this could still be very important for minimotif recognition as exemplified by the following approximation. Assuming a typical protein has a 100 nM concentration in a mammalian cell with a volume of 4 nL, then the cell contains about 240,000 molecules of this protein. Structural techniques such as NMR or X-ray crystallography are extremely insensitive requiring >10^10^ molecules for typical structure determination. If 1% of a typical protein in a cell is structured, this would amount to 2,400 molecules. Since any spectroscopic or structure determination methods do not readily detect the presence of structure in 1% of molecules, current techniques cannot be used to claim that a protein does not have structure. They can claim that most of the protein does not have structure, however, the example calculation reveals that while the presence of structure is not detectable, ∼2,400 of 240,000 protein molecules in a cell may be structured, which could certainly play a role in the recognition of minimotifs, especially if exchange between different structured and unstructured states were rapid.

A second possibility is that minimotifs located within IUPs may be in regions with stable secondary structures. We favor this hypothesis for a significant fraction of minimotifs for several reasons. Many studies support residual secondary structure in proteins that are designated as IUPs [11], [62]–[64], [76]–[82]. There are many studies since the late 1960s that identify residual structure in peptides and IUPs that are thermally or chemically denatured [67], [76], [79], [83]–[88]. Some of these secondary structure conformations are commonly found within minimotifs [23], [68], [69], [89]–[92]. For example, the disordered C-terminus of RNA polymerase II has a propensity to from polyproline and β-turn structures within known minimotif ligands [11]. The native IUP has a different structure than that observed by chemical denaturation. Likewise, the intrinsically disordered region of Neuroligin 3 becomes even more unstructured in denaturing conditions [78]. Regions of proteins between globular domains are called linkers and are often classified as IUP by disorder prediction algorithms. However, when structures of multidomain proteins are solved, these linkers, as wells as linkers containing minimotifs often have well defined secondary structures as exemplified in the structures of Src and CrkII [93], [94]. Furthermore, linkers are known to have secondary structure elements such as left-handed polyproline II helices [95]. Finally, there are many structures of minimotif peptides with secondary structures bound to their targets, e.g. [46], [47], [96], [97].

A third possibility is that minimotifs in IUP are in dynamic regions that have a high propensity to form one or more specific secondary structures and often sample these conformations. In support of this idea, a region in the C-terminus of p53 binds to 4 different proteins (S100β, Sirtuin, CBP, and Cyclin A2) with these p53 minimotifs having different secondary structures [98]. In addition to our example of RGD minimotifs binding integrins, three similar examples have been noted [99]. One explanation is that different minimotif targets select a specific structure from an ensemble of multiple structures. The formation of such secondary structures seems to be highly dependent on amino acid substitutions where even single point mutations alter secondary structures [90], [100].

Each of the aforementioned possibilities is likely to play some role in minimotif recognition. While it is not yet clear which of the three possibilities for minimotif structure recognition is most prevalent, in any case, minimotifs do binds targets in a structured manner and this is why it is important to include structure as part of the minimotif definition. In the future, the minimotif model will likely need to be adapted to include the above structural possibilities.

### Monomorphic and polymorphic: two proposed classes of minimotifs

One possibility is that there are two general classes of minimotifs that have differing thermodynamic properties and serve fundamentally different functions in cells. Minimotifs found in globular domains are structured in a fixed state that matches the binding site of its target. These *monomorphic* minimotifs should have minimal entropic penalty upon binding and are likely easily recognizable by a target upon a molecular collision. Likewise, those minimotifs that are present in an IUP and have a stable secondary structure fit into this *monomorphic* minimotif class. These minimotifs play a role by helping molecules recognize each other upon a molecular collision. Our analysis of minimotif order presented in **Fig. 5** and **Fig. S5** suggests that at least one-quarter of minimotifs are structured and fall into this class. A percentage of the ∼50% of minimotifs in the hybrid class, which is not reliable because of predication algorithm limitations, could also be *monomorphic*.

The other classes of minimotifs are those that are present in completely unstructured IUPs, assume transient secondary structures, or sample multiple secondary structures (designated *polymorphic* minimotif). Upon binding, these minimotifs have similar enthalpy to those in the *monomorphic* class, but have an entropic penalty that must be overcome to bind the target. These minimotifs would not be as easily recognized by collision with a target as a monomorphic minimotif. Thus, these *polymorphic* minimotifs would likely serve different functions, like enhancing affinity once two molecules have been recognized through an interaction with another *monomorphic* minimotifs or domain-domain interaction. Alternatively, this class could allow one region of a protein to bind multiple different targets as observed for the C-terminus of p53 [98]. It is quite possible that current blending the two classes of minimotifs together may be another source of false positive minimotif predictions. In the future, this facet may need to be considered in the minimotif model.

### Advances in minimotif model and prediction

Until now minimotifs have been considered to have sequences and functions. In this paper we present significant advancements and a revised model (**Fig. 6**) to help standardize minimotif definitions and to help reduce false positive predictions. We report fundamental flaws in the routinely used consensus protein sequence definitions as proposed at the Seefeld Convention and used in ProSite syntax [1], [101]. PSSMs have similar problems of over interpretation, ambiguity, and loss of string information. We have used sequence lexical sets for contact residues in structures, which help to solve these problems, at least in the cases examined herein. The use of computers makes the implementation of minimotif lexical sets feasible.

We propose to add structure to part of the minimotif definition. This new minimotif definition is an advance in the theoretical understanding of minimotifs and will likely help us better understand the basis of the specificity of protein interaction and posttranslational modification events in the cell.

## Materials and Methods

### Minimotifs in secondary structure

To determine the types of secondary structures for minimotifs, we gathered examples of the 32 types of secondary structure from the literature. We then ran structural comparisons of the examples of the 32 secondary structures and generated a score variability matrix for the structures. We were then able to use this variability data to compare structures of minimotif instances to each secondary structure, and thus assign each putative minimotif a secondary structure based on its closest match using the Centroid Algorithm, assuming the match did not exceed the variability of the structure. If the closest match to a putative minimotif instance exceeded the variability threshold, the minimotif was instead assigned to the “random coil” category.

### Workflow to identify minimotif structural matches in the PDB

A workflow for identification of structured lexica in the PDB is provided in **Fig. S6**. As a preliminary step, the literature was examined thoroughly to gather information on all known secondary structures. 32 such structures with examples exist in the available literature; one additional structure exists but without an example (the δ-turn), and there is one category for structures that do not fit into the 32 secondary structures, designated “random coil.” All known examples of each type of secondary structure were entered into a table in a MySQL (http://www.mysql.com) database. The latest version of the Protein Data Bank (PDB, http://www.rcsb.org) was converted into a MySQL database and then searched to determine which examples had complete structural information available (defined as “for every residue in the example, there must exist experimentally-determined 3D coordinates for at least the C_α_ atom, and preferably for all backbone and side chain atoms other than hydrogen”). Those examples with complete structural information available were listed in a second MySQL table; a combined table of secondary structures with complete secondary structural information was then generated by joining the two tables. The known examples of each secondary structure (as defined in the literature) were then compared to each other using a custom-written Java program utilizing the Centroid Algorithm; the maximum score obtained during this comparison was used as the limit of variation for each secondary structure, resulting in a canonical version of each secondary structure to be used for comparison.

For each minimotif in a list taken from the Minimotif Miner 3.0 data set, the following procedure was then performed.

The known positive instances of each minimotif were compared using the customized Centroid Algorithm for structural comparison, and to establish variation limits for that minimotif. The PDB was then searched for all minimotif sequence matches, and this list was then pruned to include only those sequence matches for which complete structural information was available (using the same definition of “complete structural information” given above for the secondary structure examples). Each instance with complete structural information was then compared to the canonical instance of each secondary structure, using a custom-written Java program based upon the Centroid Algorithm. The lowest score was taken and compared to the variation limits for the secondary structure and to the variation limits established for the minimotif itself, using the same Centroid Algorithm-based Java program. If the lowest score was higher than these limits, this instance of the minimotif sequence was determined to be in random coil configuration. Otherwise, it was determined to be in the secondary structure with the lowest score. The resulting structure, score, instance sequence, and other data about the minimotif sequence match were then saved to an additional table in the MySQL database.

### Centroid structural comparison algorithm

For the structural comparisons, we used the Centroid Algorithm, a modified version of the Kundeti/Rajasekaran Center-of-Gravity algorithm for comparing structures [102]. This algorithm was chosen for its speed. The general process for the algorithm is as follows:

1. Centroid calculation
  - Find the 3-dimensional centroid of all atoms in the first structure to be compared.
  - Find the 3-dimensional centroid of all atoms in the second structure to be compared.
2. Distance from centroid
  - For each atom in the first structure, find the distance to the centroid. Store these values in a vector (V_1_).
  - For each atom in the second structure, find the distance to the centroid. Store these values in a vector (V_2_).
3. Sorting
  - Sort the values in V_1_ from smallest to largest.
  - Sort the values in V_2_ from smallest to largest.
4. Summation
  - Calculate the differences between vectors V_1_ and V_2_ at each position
  - Multiply the difference between the vectors at each position by a weighting factor inversely proportional to its position in the vector (particles farther from the centroid are increased in significance).
  - Calculate the sum of the weighted differences.
  - (Optional) Divide by a normalization factor, if required. This step should be used if scores for structures containing different numbers of atoms are to be directly compared. If the output is to be a binary result, or if numeric results do not need to be compared directly to scores for other structures with different numbers of atoms, this normalization step is not required.
5. Result
  - If a numeric result is desired, return the sum from step 4.
  - If a binary result is desired (“match” or “no match”), compare the sum from step 4 to a pre-determined error threshold ε. If the sum exceeds ε, return “no match.” Otherwise, return “match.”

### Generation of figures

Protein structure figures were created using Jmol (http://www.jmol.org), PyMol (http://pymol.org), and MolMol (http://www-theor.ch.cam.ac.uk/IT/software/molmol.html ) [103]–[105]. Some PDB data parsing and protein sequence creation was performed using BioJava 3 (http://www.biojava.org) [106].

## Supporting Information

Figure S1 **Gallery of structures for different motifs (blue) bound to their respective domain partners.** Domain names and PDB identifiers are shown.
(PDF)

Figure S2 **xYxN Lexica.** Lexica of xYxN that are observed to form the correct structure. The 91 lexica of consensus sequence xYxN that are observed to form the correct structure (β-turn type I) in nature are colored green.
(PDF)

Figure S3 **Graph of lexical specificity of xYxN.** Plot of the normalized number of occurrences in β-turn type I. The depth and horizontal axes show the single letter IUPAC code for amino acids in the x_1_ and x_2_ position of the xYxN consensus minimotif, respectively. Colored labeled bars indicate lexica where a known structure of a complex of the Grb2 SH2 domain with this peptide sequence exists in the PDB.
(PDF)

Figure S4 A. Distribution of minimotif secondary structure folds in the PDB. Bar graph showing the number of occurrences of IxxNT (**A**), [RK]xxK (**B**), PxxPxK (**C**), xx[ST]x[IVL]>(**D**), and [ILV]QxxxRGxxx[RK] (**E**) sequences from the PDB in each type of secondary structure. Arrows indicates the correct structure of known ligands.
(PDF)

Figure S5 **Minimotif order/disorder prediction statistics.** A. Pie graphs for 8 types of modification minimotifs show the different prevalence of hybrid, structured, and unstructured minimotifs. **B**. PONDR VLXT disorder prediction results for all motif activity classes with more than 100 instances.
(PDF)

Figure S6 **General workflow for identifying minimotifs with the correct minimotifs structure.**
(PDF)

Table S1 Known positives that bind to Grb2 SH2 domain. Rows where the lexical sequence of the known positive was successfully predicted to be in the correct structure are colored green. 26 of the 29 sequences were found in the correct structure, a success rate of 89.7%. The 29 known positive instance minimotifs consist of a total of 22 distinct lexical sequences, of which 19 were found in the correct structure, a success rate of 86.4%.
(PDF)

Reference List S1 (PDF)
