# Supplementary material for: Secondary Structure, a Missing Component of Sequence-Based Minimotif Definitions
Source: PLoS One. 2012 Dec 7;7(12):e49957. doi: 10.1371/journal.pone.0049957 (PMC3517595; doi:10.1371/journal.pone.0049957)
Supplement: Figure S1 — Gallery of structures for different motifs (blue) bound to their respective domain partners. Domain names and PDB identifiers are shown. (PDF) [file pone.0049957.s001.pdf]

SUPPLEMENTARY DATA

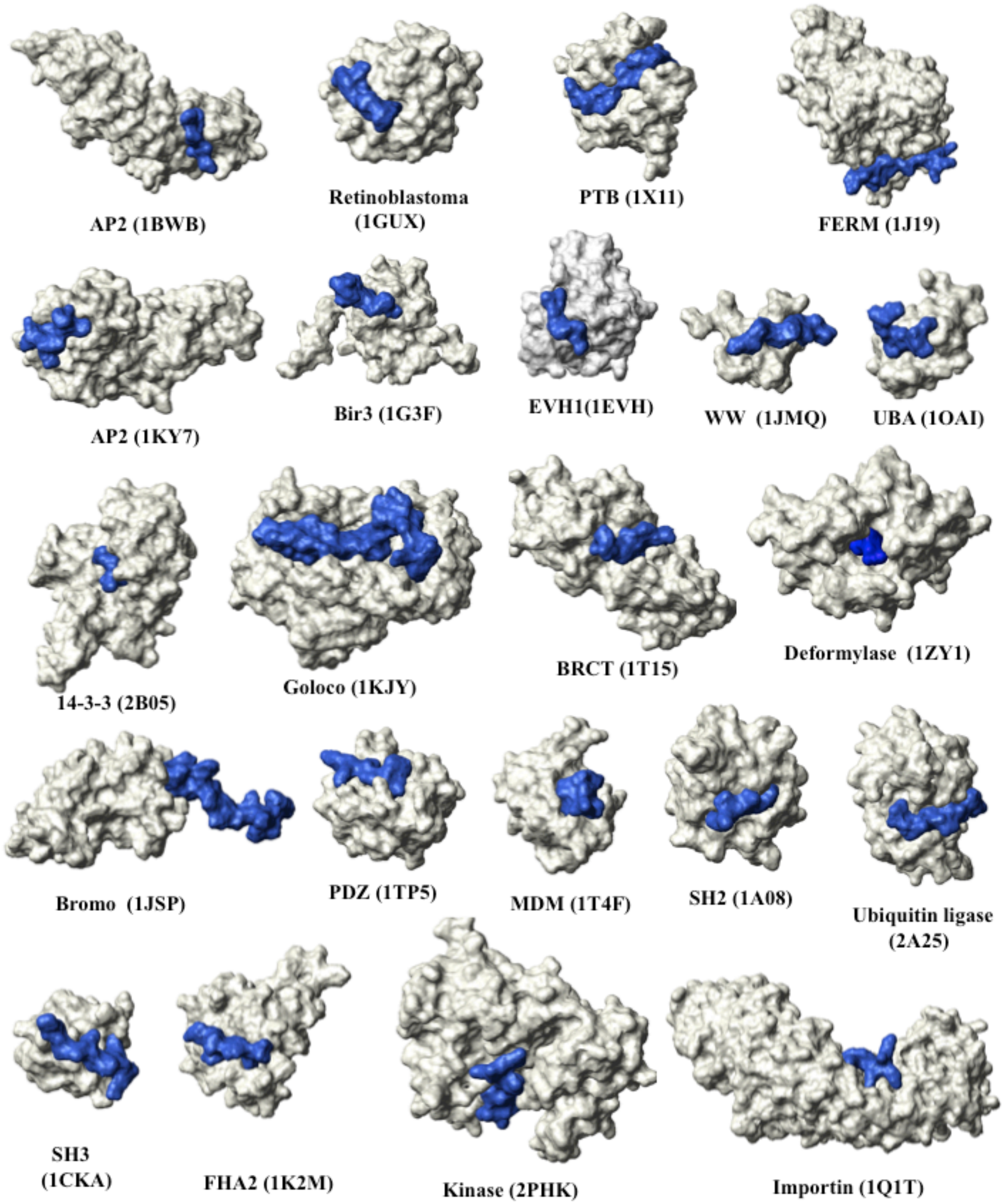

Figure S1. Gallery of structures for different motifs (blue) bound to their respective domain partners. Domain names and PDB identifiers are shown.
