# Supplementary material for: Secondary Structure, a Missing Component of Sequence-Based Minimotif Definitions
Source: PLoS One. 2012 Dec 7;7(12):e49957. doi: 10.1371/journal.pone.0049957 (PMC3517595; doi:10.1371/journal.pone.0049957)
Supplement: Figure S2 — xYxN Lexica. Lexica of xYxN that are observed to form the correct structure. The 91 lexica of consensus sequence xYxN that are observed to form the correct structure (β-turn type I) in nature are colored green. (PDF) [file pone.0049957.s002.pdf]

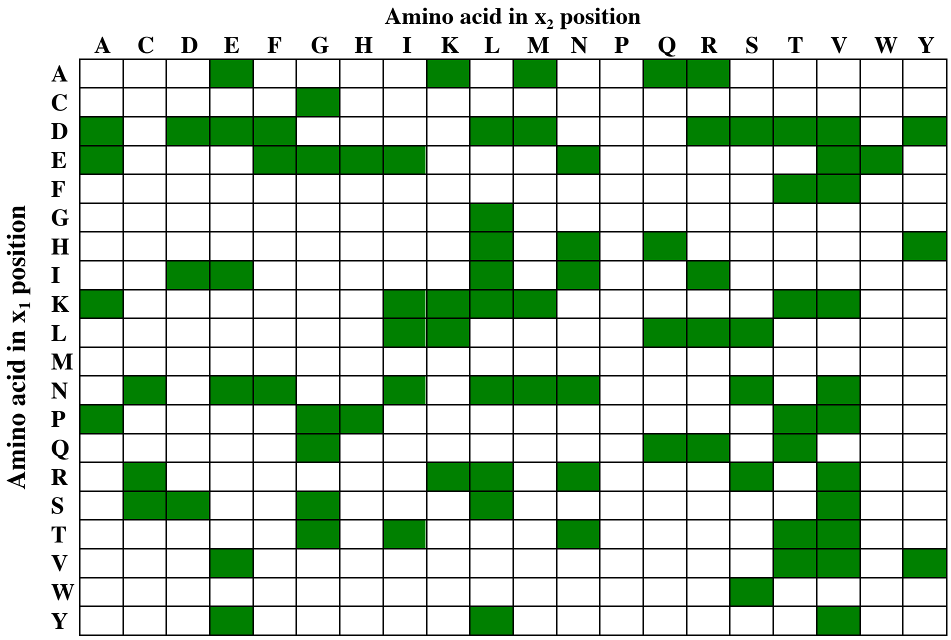

Figure S2. xYxN Lexica. Lexica of xYxN that are observed to form the correct structure. The 91 lexica of consensus sequence xYxN that are observed to form the correct structure (β-turn type I) in nature are colored green.
