# Supplementary material for: Secondary Structure, a Missing Component of Sequence-Based Minimotif Definitions
Source: PLoS One. 2012 Dec 7;7(12):e49957. doi: 10.1371/journal.pone.0049957 (PMC3517595; doi:10.1371/journal.pone.0049957)
Supplement: Figure S3 — Graph of lexical specificity of xYxN. Plot of the normalized number of occurrences in β-turn type I. The depth and horizontal axes show the single letter IUPAC code for amino acids in the x1 and x2 position of the xYxN consensus minimotif, respectively. Colored labeled bars indicate lexica where a known structure of a complex of the Grb2 SH2 domain with this peptide sequence exists in the PDB. (PDF) [file pone.0049957.s003.pdf]

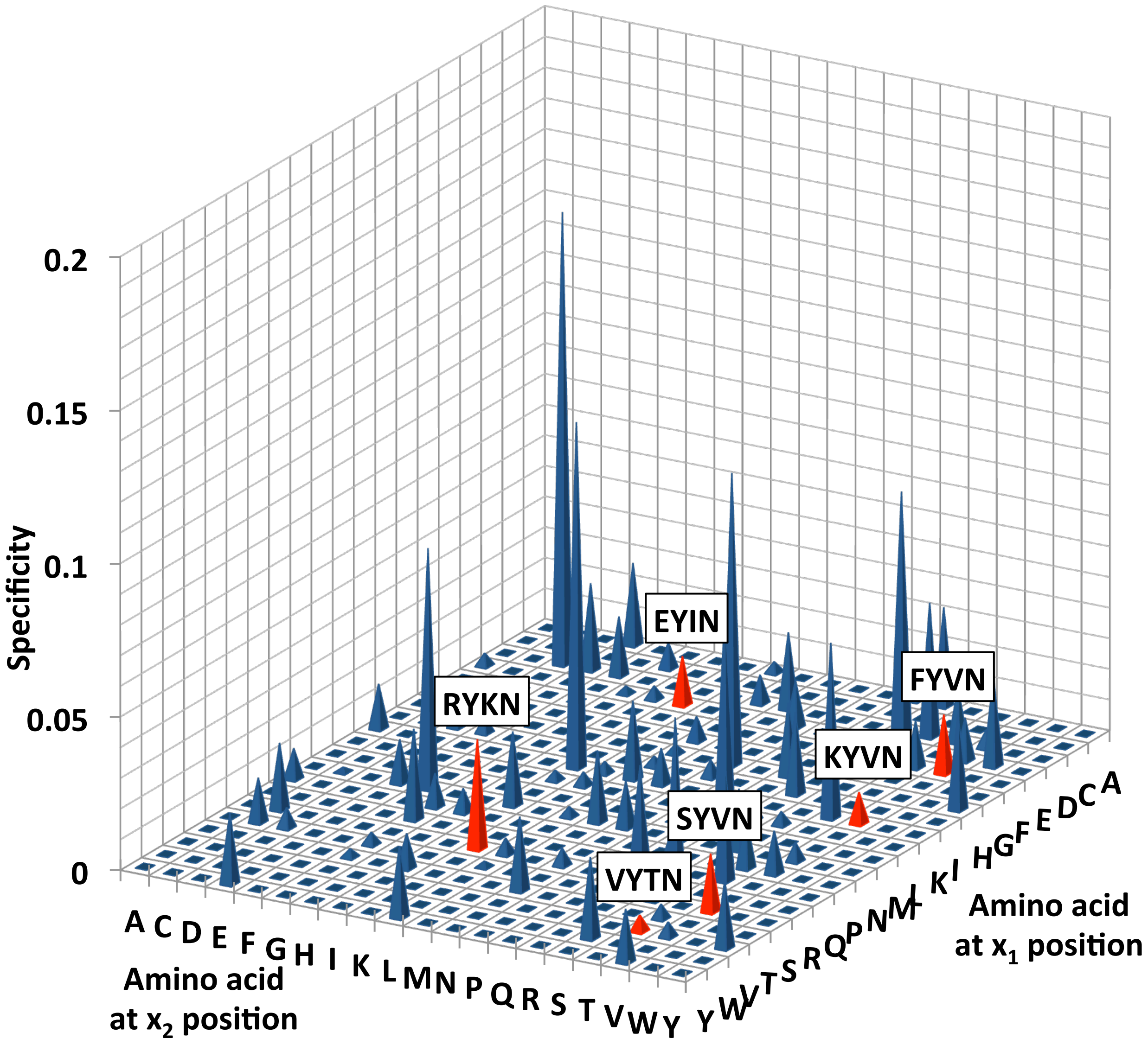

Figure S3. Graph of lexical specificity of xYxN. Plot of the normalized number of occurrences in β-turn type I. The depth and horizontal axes show the single letter IUPAC code for amino acids in the x_1 and x_2 position of the xYxN consensus minimotif, respectively. Colored labeled bars indicate lexica where a known structure of a complex of the Grb2 SH2 domain with this peptide sequence exists in the PDB.
