# Supplementary material for: Secondary Structure, a Missing Component of Sequence-Based Minimotif Definitions
Source: PLoS One. 2012 Dec 7;7(12):e49957. doi: 10.1371/journal.pone.0049957 (PMC3517595; doi:10.1371/journal.pone.0049957)
Supplement: Figure S4 — A. Distribution of minimotif secondary structure folds in the PDB. Bar graph showing the number of occurrences of IxxNT (A), [RK]xxK (B), PxxPxK (C), xx[ST]x[IVL]>(D), and [ILV]QxxxRGxxx[RK] (E) sequences from the PDB in each type of secondary structure. Arrows indicates the correct structure of known ligands. (PDF) [file pone.0049957.s004.pdf]

A

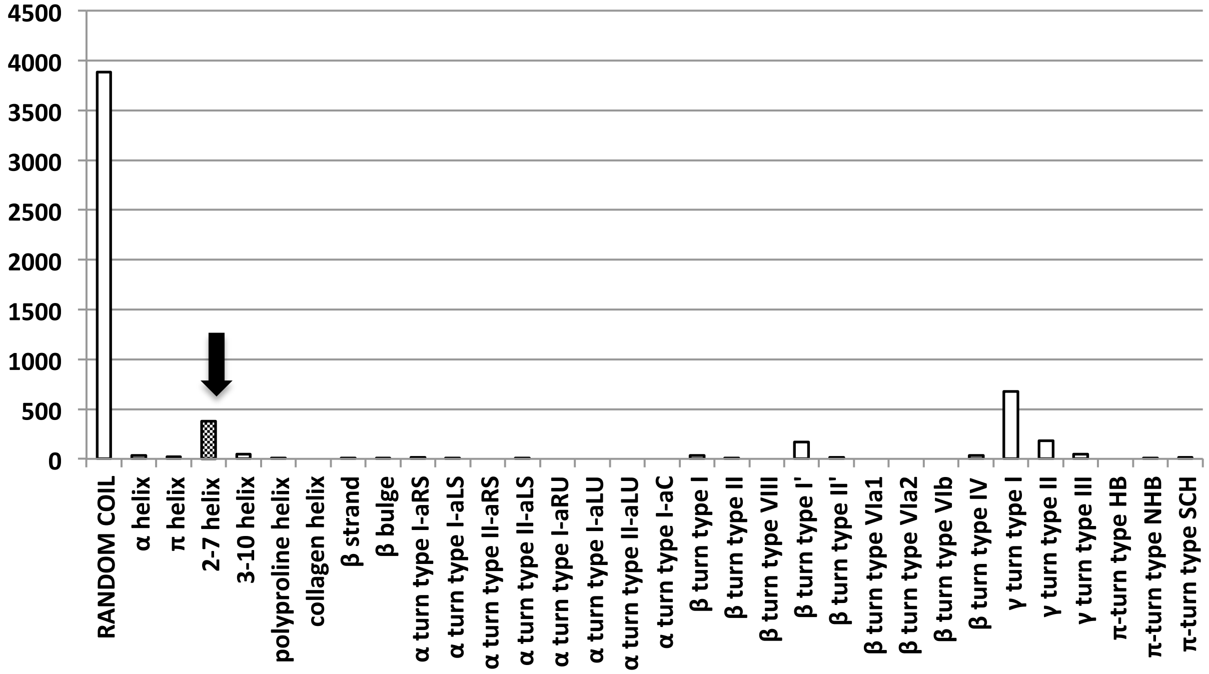
4500
4000
3500
3000
2500
2000
1500
1000
500
0
RANDOM COIL
α helix
π helix
2-7 helix
3-10 helix
polyproline helix
collagen helix
β strand
β bulge
α turn type I-aRS
α turn type I-aLS
α turn type II-aRS
α turn type II-aLS
α turn type I-aRU
α turn type I-aLU
α turn type II-aLU
α turn type I-aC
β turn type I
β turn type II
β turn type VIII
β turn type I'
β turn type II'
β turn type VIa1
β turn type VIa2
β turn type VIb
β turn type IV
γ turn type I
γ turn type II
γ turn type III
π-turn type HB
π-turn type NHB
π-turn type SCH

B

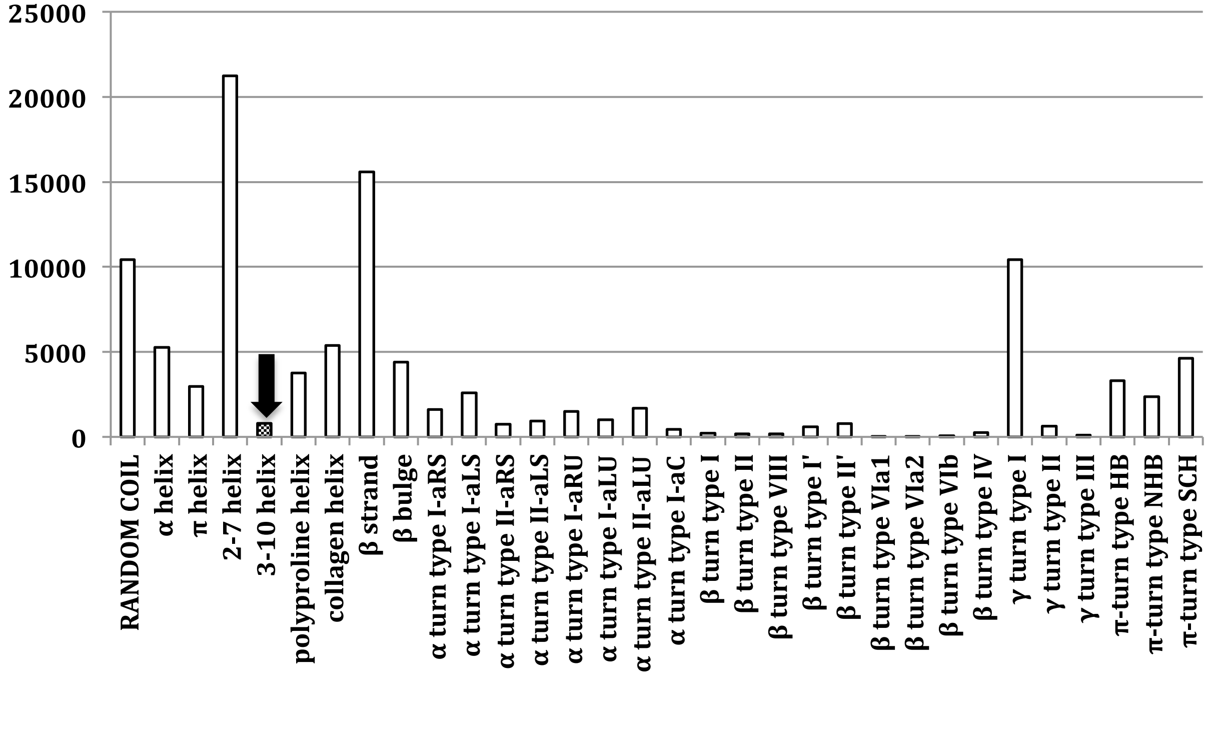
25000
20000
15000
10000
5000
0
RANDOM COIL
α helix
π helix
2-7 helix
3-10 helix
polyproline helix
collagen helix
β strand
β bulge
α turn type I-aRS
α turn type I-aLS
α turn type II-aRS
α turn type II-aLS
α turn type I-aRU
α turn type I-aLU
α turn type II-aLU
α turn type I-aC
β turn type I
β turn type II
β turn type VIII
β turn type I'
β turn type II'
β turn type VIa1
β turn type VIa2
β turn type VIb
β turn type IV
γ turn type I
γ turn type II
γ turn type III
π-turn type HB
π-turn type NHB
π-turn type SCH

C

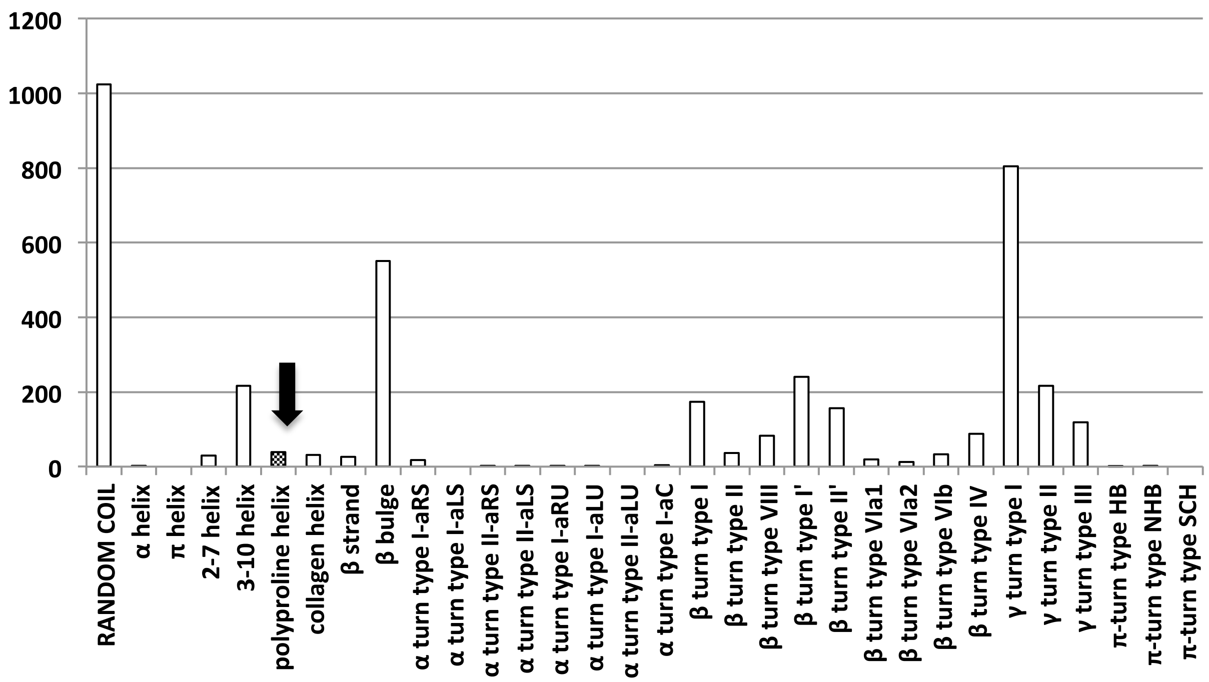
1200
1000
800
600
400
200
0
RANDOM COIL
α helix
π helix
2-7 helix
3-10 helix
polyproline helix
collagen helix
β strand
β bulge
α turn type I-aRS
α turn type I-aLS
α turn type II-aRS
α turn type II-aLS
α turn type I-aRU
α turn type I-aLU
α turn type II-aLU
α turn type I-aC
β turn type I
β turn type II
β turn type VIII
β turn type I'
β turn type II'
β turn type VIa1
β turn type VIa2
β turn type VIb
β turn type IV
γ turn type I
γ turn type II
γ turn type III
π-turn type HB
π-turn type NHB
π-turn type SCH

D

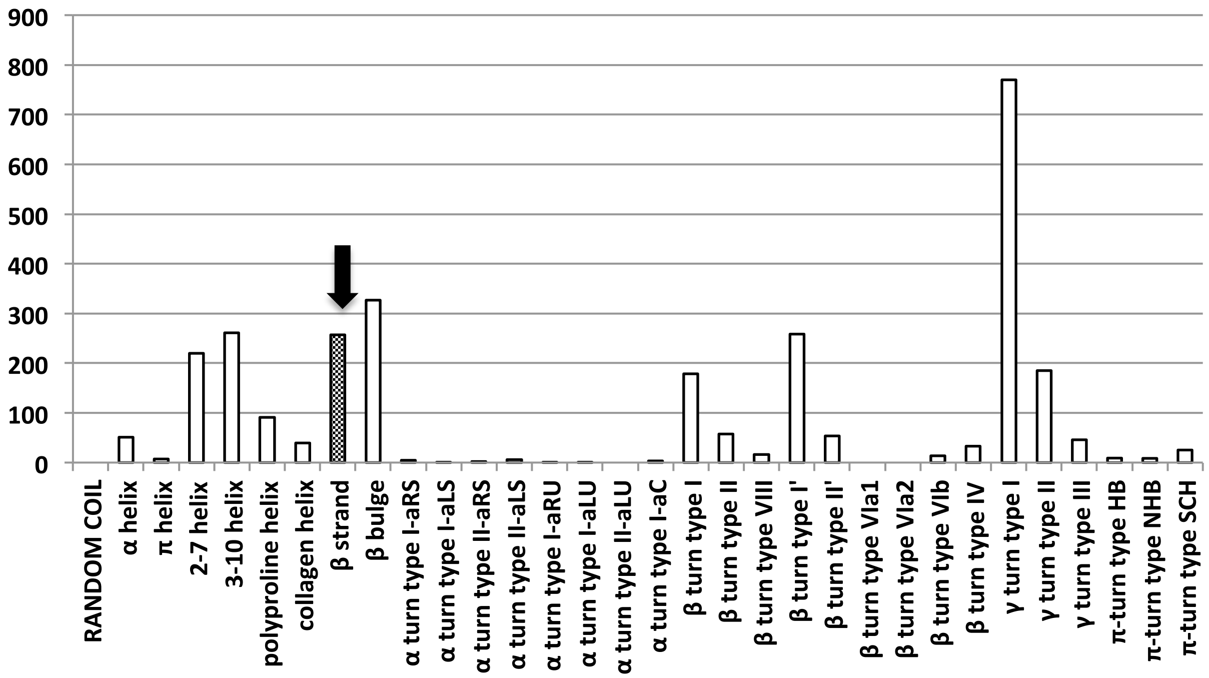
900
800
700
600
500
400
300
200
100
0
RANDOM COIL
α helix
π helix
2-7 helix
3-10 helix
polyproline helix
collagen helix
β strand
β bulge
α turn type I-aRS
α turn type I-aLS
α turn type II-aRS
α turn type II-aLS
α turn type I-aRU
α turn type I-aLU
α turn type II-aLU
α turn type I-aC
β turn type I
β turn type II
β turn type VIII
β turn type I'
β turn type II'
β turn type VIa1
β turn type VIa2
β turn type VIb
β turn type IV
γ turn type I
γ turn type II
γ turn type III
π-turn type HB
π-turn type NHB
π-turn type SCH

E

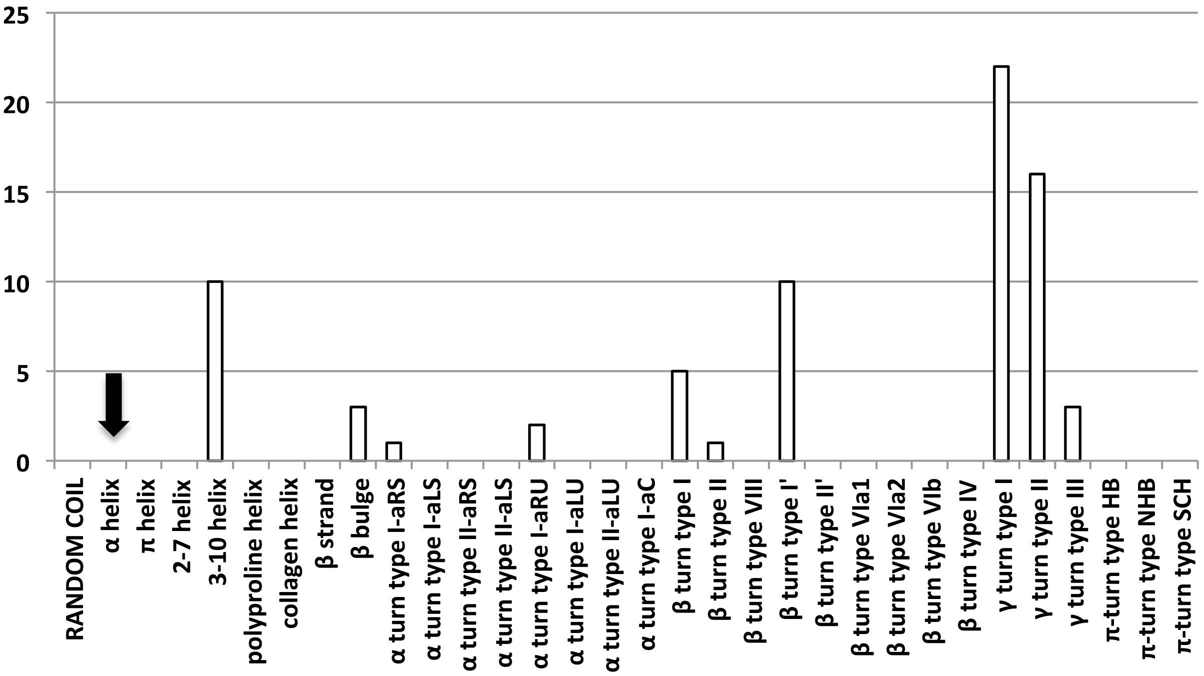

Figure S4 A. Distribution of minimotif secondary structure folds in the PDB. Bar graph showing the number of occurrences of IxxNT (**A**), [RK]xxK (**B**), PxxPxK (**C**), xx[ST]x[IVL]> (**D**), and [ILV]QxxxRGxxx[RK] (**E**) sequences from the PDB in each type of secondary structure. Arrows indicates the correct structure of known ligands
