# Supplementary material for: Secondary Structure, a Missing Component of Sequence-Based Minimotif Definitions
Source: PLoS One. 2012 Dec 7;7(12):e49957. doi: 10.1371/journal.pone.0049957 (PMC3517595; doi:10.1371/journal.pone.0049957)
Supplement: Figure S5 — Minimotif order/disorder prediction statistics. A. Pie graphs for 8 types of modification minimotifs show the different prevalence of hybrid, structured, and unstructured minimotifs. B. PONDR VLXT disorder prediction results for all motif activity classes with more than 100 instances. (PDF) [file pone.0049957.s005.pdf]

A

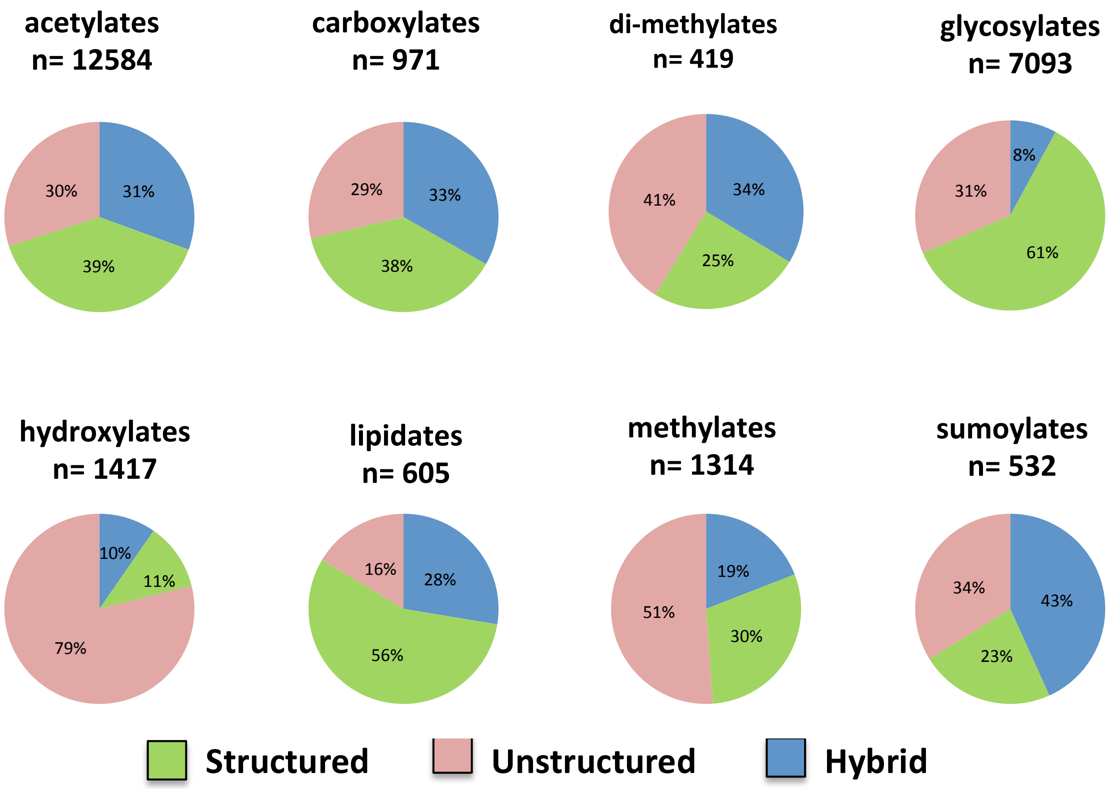

B

Motif Activity	Hybrid	Structured	Unstructured	Total
phosphorylates	54066	30736	92153	**176955**
modifies motif(s)	4265	16714	4170	**25149**
acetylates	3848	4971	3765	**12584**
proteolyzes	2466	4374	2005	**8845**
glycosylates	563	4298	2232	**7093**
binds (general)	509	566	1072	**2147**
n-glycolysates	458	1423	155	**2036**
hydroxylates	137	162	1118	**1417**
methylates	251	391	672	**1314**
carboxylates	323	370	278	**971**
ubiquitinates	323	240	160	**723**
lipidates	167	339	99	**605**
sumoylates	230	122	180	**532**
O-GlcNAcates	157	73	191	**421**
di-methylates	141	105	173	**419**
mono-methylates	72	54	108	**234**
sulfonates	55	74	23	**152**
**Total**	**68031**	**65012**	**108554**	**241597**

Figure S5. Minimotif order/disorder prediction statistics. A. Pie graphs for 8 types of modification minimotifs show the different prevalence of hybrid, structured, and unstructured minimotifs. **B**. PONDR VLXT disorder prediction results for all motif activity classes with more than 100 instances.
