# Supplementary figures and images for: Secondary Structure, a Missing Component of Sequence-Based Minimotif Definitions
Source: PLoS One. 2012 Dec 7;7(12):e49957. doi: 10.1371/journal.pone.0049957 (PMC3517595; doi:10.1371/journal.pone.0049957)

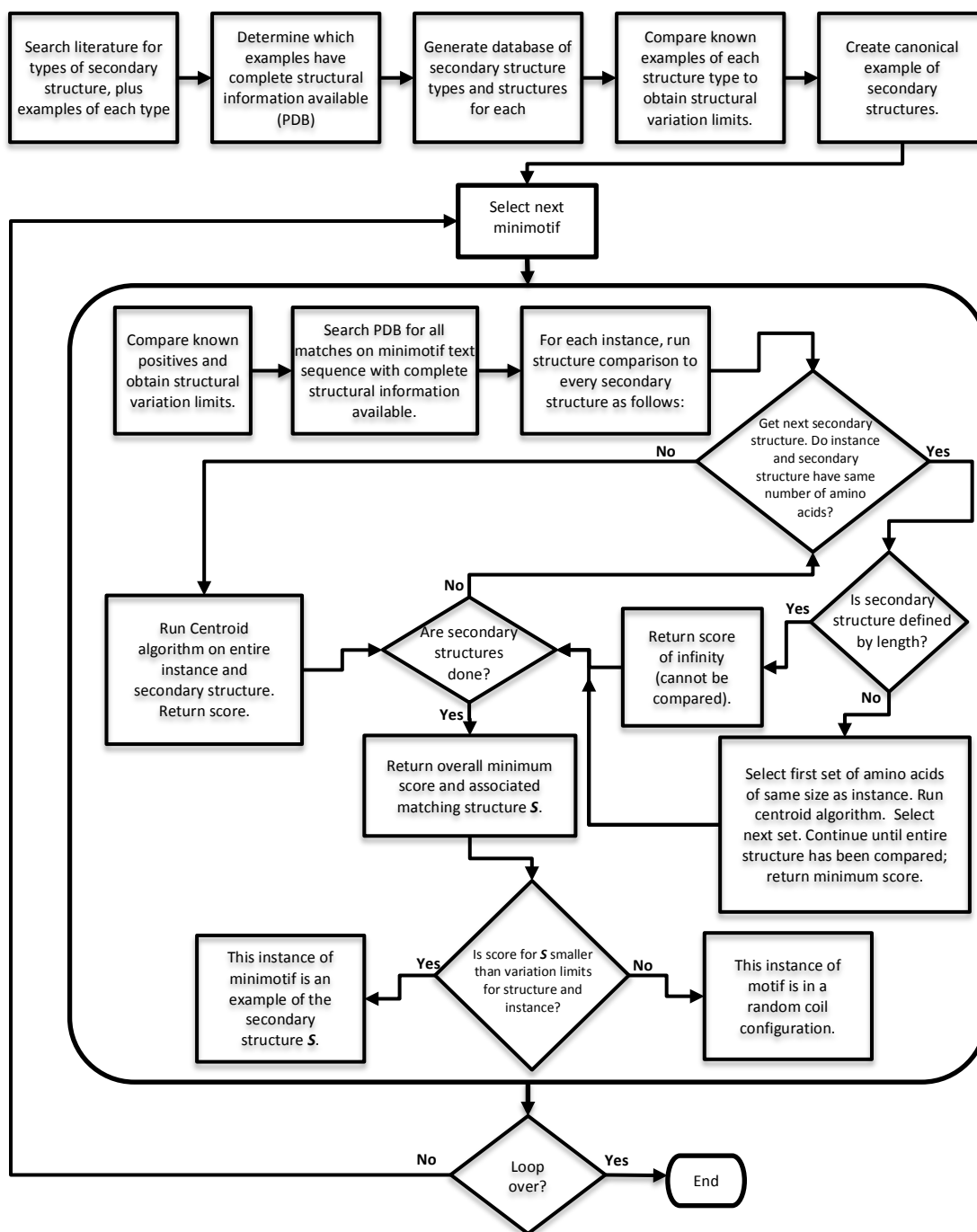

**Figure S6. General workflow for identifying minimotifs with the correct minimotifs structure.**

Supplement: Figure S6 — General workflow for identifying minimotifs with the correct minimotifs structure. (PDF) [file pone.0049957.s006.pdf]
