# Supplementary material for: Secondary Structure, a Missing Component of Sequence-Based Minimotif Definitions
Source: PLoS One. 2012 Dec 7;7(12):e49957. doi: 10.1371/journal.pone.0049957 (PMC3517595; doi:10.1371/journal.pone.0049957)
Supplement: Table S1 — Known positives that bind to Grb2 SH2 domain. Rows where the lexical sequence of the known positive was successfully predicted to be in the correct structure are colored green. 26 of the 29 sequences were found in the correct structure, a success rate of 89.7%. The 29 known positive instance minimotifs consist of a total of 22 distinct lexical sequences, of which 19 were found in the correct structure, a success rate of 86.4%. (PDF) [file pone.0049957.s007.pdf]

Table S1. Known positives that bind to Grb2 SH2 domain.

Interacting protein	Accession number	Y Residue	Sequence	Predicted	Reference
SHC1	NP_003020	239	PPDHQ**YYN**DFPGK	No	(Velazquez et al., 2000)
SHC1	NP_003020	318	FDDPS**YVN**VQNLD	Yes	(Skolnik et al., 1993)
EGFR	NP_005219	1191	LPVPE**YIN**QSVPK	Yes	(Rahuel et al., 1998)
IRS-1	NP_005535	895	KSPGE**YVN**IEFGS	Yes	(Skolnik et al., 1993)
FAK	NP_032008	925	SNDKV**YEN**VTGLV	Yes	(Schlaepfer and Hunter, 1996)
PDGFRb	NP_002600	716	PSAEL**YSN**ALPVG	Yes	(Arvidsson et al., 1994)
Nicotinic Rδ	NP_000742	393	SKAQE**YFN**IKSRS	Yes	(Colledge and Froehner, 1997)
BCR	NP_004318	177	AEKPF**YVN**VEFHH	Yes	(Rahuel et al., 1996)
SHP-1	NP_536858	538	GQESE**YGN**ITYPP	Yes	(Kon-Kozlowski et al., 1996)
SHP-2	NP_002825	584	DSARV**YEN**VGLMQ	Yes	(Vogel and Ullrich, 1996)
FRS-2	NP_006645	306	VNKLV**YEN**INGLS	Yes	(Kouhara et al., 1997)
FRS-2	NP_006645	339	TALLN**YEN**LPSLP	Yes	(Kouhara et al., 1997)
CD19	P15391	330	GPQNQ**YGN**VLSLP	Yes	(Brooks et al., 2000)
CD19	P15391	360	GTAPS**YGN**PSSDV	Yes	(Brooks et al., 2000)
RET	NP_066124	1096	PNDSV**YAN**WMLSP	No	(Alberti et al., 1998); (Liu et al., 1996)
MET	NP_000236	1356	HVNAT**YVN**VKCVA	Yes	(Schiering et al., 2000)
RPTPε	NP_006495	695	DIFSD**YAN**FK>	Yes	(Toledano-Katchalski and Elson, 1999)
RPTPα	NP_543031	789	DAFSD**YAN**FK>	Yes	(den Hertog et al., 1994)
ErbB2	NP_058699	1139	SPQPE**YVN**QPDVR	Yes	(Ricci et al., 1995)
LAT	NP_034819	175	ESCED**YVN**VPESE	Yes	(Zhang et al., 2000)
LAT	NP_034819	195	DGSRE**YVN**VSPEQ	Yes	(Zhang et al., 2000)
LAT	NP_034819	235	EEAPD**YEN**LQELN	Yes	(Zhang et al., 2000)
VEGFR1	NP_002010	1213	SDDVR**YVN**AFKFM	Yes	(Ito et al., 1998)
cMET	NP_000236	1356	HVNAT**YVN**VKCVA	Yes	(Fournier et al., 1996)
cKit	NP_000213	936	STNHI**YSN**LANCS	No	(Thömmes et al., 1999)
cKit	NP_000213	703	AEAAL**YKN**LLHSK	Yes	(Thömmes et al., 1999)
BTLA	NP_861445	226	SETGI**YDN**DPDLC	Yes	(Gavrieli and Murphy, 2006)
Tek	NP_000450	1100	EERKT**YVN**TTLYE	Yes	(Jones et al., 1999)
ETV6	NP_001978	314	REDLA**YMN**HIMVS	Yes	(Million et al., 2004)

Rows where the lexical sequence of the known positive was successfully predicted to be in the correct structure are colored green. 26 of the 29 sequences were found in the correct structure, a success rate of 89.7%. The 29 known positive instance minimotifs consist of a total of 22 distinct lexical sequences, of which 19 were found in the correct structure, a success rate of 86.4%.
