# Supplementary material for: Secondary Structure, a Missing Component of Sequence-Based Minimotif Definitions
Source: PLoS One. 2012 Dec 7;7(12):e49957. doi: 10.1371/journal.pone.0049957 (PMC3517595; doi:10.1371/journal.pone.0049957)
Supplement: Reference List S1 — (PDF) [file pone.0049957.s008.pdf]

SUPPLEMENTAL REFERENCES

Alberti, L., Borrello, M.G., Ghizzoni, S., Torriti, F., Rizzetti, M.G., and Pierotti, M.A. (1998). Grb2 binding to the different isoforms of Ret tyrosine kinase. Oncogene *17*, 1079–1087.

Arvidsson, A.K., Rupp, E., Nånberg, E., Downward, J., Rönnstrand, L., Wennström, S., Schlessinger, J., Heldin, C.H., and Claesson-Welsh, L. (1994). Tyr-716 in the platelet-derived growth factor beta-receptor kinase insert is involved in GRB2 binding and Ras activation. Mol. Cell. Biol. *14*, 6715–6726.

Brooks, S.R., Li, X., Volanakis, E.J., and Carter, R.H. (2000). Systematic analysis of the role of CD19 cytoplasmic tyrosines in enhancement of activation in Daudi human B cells: clustering of phospholipase C and Vav and of Grb2 and Sos with different CD19 tyrosines. J. Immunol. *164*, 3123–3131.

Colledge, M., and Froehner, S.C. (1997). Tyrosine phosphorylation of nicotinic acetylcholine receptor mediates Grb2 binding. J. Neurosci. *17*, 5038–5045.

Fournier, T.M., Kamikura, D., Teng, K., and Park, M. (1996). Branching tubulogenesis but not scatter of madin-darby canine kidney cells requires a functional Grb2 binding site in the Met receptor tyrosine kinase. J. Biol. Chem. *271*, 22211–22217.

Gavrieli, M., and Murphy, K.M. (2006). Association of Grb-2 and PI3K p85 with phosphotyrosile peptides derived from BTLA. Biochem. Biophys. Res. Commun. *345*, 1440–1445.

den Hertog, J., Tracy, S., and Hunter, T. (1994). Phosphorylation of receptor protein-tyrosine phosphatase alpha on Tyr789, a binding site for the SH3-SH2-SH3 adaptor protein GRB-2 in vivo. EMBO J. *13*, 3020–3032.

Ito, N., Wernstedt, C., Engström, U., and Claesson-Welsh, L. (1998). Identification of vascular endothelial growth factor receptor-1 tyrosine phosphorylation sites and binding of SH2 domain-containing molecules. J. Biol. Chem. *273*, 23410–23418.

Jones, N., Master, Z., Jones, J., Bouchard, D., Gunji, Y., Sasaki, H., Daly, R., Alitalo, K., and Dumont, D.J. (1999). Identification of Tek/Tie2 binding partners. Binding to a multifunctional docking site mediates cell survival and migration. J. Biol. Chem. *274*, 30896–30905.

Kon-Kozlowski, M., Pani, G., Pawson, T., and Siminovitch, K.A. (1996). The tyrosine phosphatase PTP1C associates with Vav, Grb2, and mSos1 in hematopoietic cells. J. Biol. Chem. *271*, 3856–3862.

Kouhara, H., Hadari, Y.R., Spivak-Kroizman, T., Schilling, J., Bar-Sagi, D., Lax, I., and Schlessinger, J. (1997). A lipid-anchored Grb2-binding protein that links FGF-receptor activation to the Ras/MAPK signaling pathway. Cell *89*, 693–702.

Liu, F., Hill, D.E., and Chernoff, J. (1996). Direct binding of the proline-rich region of protein tyrosine phosphatase 1B to the Src homology 3 domain of p130(Cas). J. Biol. Chem. *271*, 31290–31295.

Million, R.P., Harakawa, N., Roumiantsev, S., Varticovski, L., and Van Etten, R.A. (2004). A direct binding site for Grb2 contributes to transformation and leukemogenesis by the Tel-Abl (ETV6-Abl) tyrosine kinase. Mol. Cell. Biol. *24*, 4685–4695.

Rahuel, J., García-Echeverría, C., Furet, P., Strauss, A., Caravatti, G., Fretz, H., Schoepfer, J., and Gay, B. (1998). Structural basis for the high affinity of amino-aromatic SH2 phosphopeptide ligands. J. Mol. Biol. *279*, 1013–1022.

Rahuel, J., Gay, B., Erdmann, D., Strauss, A., GarciaEcheverria, C., Furet, P., Caravatti, G., Fretz, H., Schoepfer, J., and Grutter, M.G. (1996). Structural basis for specificity of GRB2-SH2 revealed by a novel ligand binding mode. Nature Structural.Biol. *3*, 586–589.

Ricci, A., Lanfrancone, L., Chiari, R., Belardo, G., Pertica, C., Natali, P.G., Pelicci, P.G., and Segatto, O. (1995). Analysis of protein-protein interactions involved in the activation of the Shc/Grb-2 pathway by the ErbB-2 kinase. Oncogene *11*, 1519–1529.

Schiering, N., Casale, E., Caccia, P., Giordano, P., and Battistini, C. (2000). Dimer formation through domain swapping in the crystal structure of the Grb2-SH2-Ac-pYVNV complex. Biochemistry *39*, 13376–13382.

Schlaepfer, D.D., and Hunter, T. (1996). Evidence for in vivo phosphorylation of the Grb2 SH2-domain binding site on focal adhesion kinase by Src-family protein-tyrosine kinases. Mol. Cell. Biol. *16*, 5623–5633.

Skolnik, E.Y., Lee, C.H., Batzer, A., Vicentini, L.M., Zhou, M., Daly, R., Myers, M.J., Jr, Backer, J.M., Ullrich, A., and White, M.F. (1993). The SH2/SH3 domain-containing protein GRB2 interacts with tyrosine-phosphorylated IRS1 and Shc: implications for insulin control of ras signalling. EMBO J. *12*, 1929–1936.

Thömmes, K., Lennartsson, J., Carlberg, M., and Rönnstrand, L. (1999). Identification of Tyr-703 and Tyr-936 as the primary association sites for Grb2 and Grb7 in the c-Kit/stem cell factor receptor. Biochem. J. *341 (Pt 1)*, 211–216.

Toledano-Katchalski, H., and Elson, A. (1999). The transmembranal and cytoplasmic forms of protein tyrosine phosphatase epsilon physically associate with the adaptor molecule Grb2. Oncogene *18*, 5024–5031.

Velazquez, L., Gish, G.D., van Der Geer, P., Taylor, L., Shulman, J., and Pawson, T. (2000). The shc adaptor protein forms interdependent phosphotyrosine-mediated protein complexes in mast cells stimulated with interleukin 3. Blood *96*, 132–138.

Vogel, W., and Ullrich, A. (1996). Multiple in vivo phosphorylated tyrosine phosphatase SHP-2 engages binding to Grb2 via tyrosine 584. Cell Growth Differ. *7*, 1589–1597.

Zhang, W., Trible, R.P., Zhu, M., Liu, S.K., McGlade, C.J., and Samelson, L.E. (2000). Association of Grb2, Gads, and phospholipase C-gamma 1 with phosphorylated LAT tyrosine residues. Effect of LAT tyrosine mutations on T cell angigen receptor-mediated signaling. J. Biol. Chem. *275*, 23355–23361.
